# A facile and efficient route to one-pot synthesis of new cyclophanes using vinamidinium salts†

**DOI:** 10.1039/d0ra10548a

**Published:** 2021-04-13

**Authors:** Nooshin Golzar, Abdolmohammad Mehranpour, Najmeh Nowrouzi

**Affiliations:** Department of Chemistry, Faculty of Sciences, Persian Gulf University Bushehr 75169 Iran ammehranpour@hotmail.com

## Abstract

In this study, an efficient method for the synthesis of new cyclophanes (5a–f, 6a–g) through the condensation of 1,4-phenylenedimethanamine (3) or 2,3,5,6-tetramethylbenzene-1,4-diamine (4) with 2-substituted vinamidiniums (2a–g) is described. The cyclophane derivatives are obtained in good to excellent yields in the presence of acetic acid in refluxing acetonitrile after 15 h. The structure of new compounds was validated based on their spectral data (^1^H NMR, ^13^C NMR, IR) and elemental analysis.

## Abstract 2

New cyclophane derivatives are synthesized from the reaction of γ-substituted vinamidinium salts with 1,4-phenylenedimethanamine and 2,3,5,6-tetramethylbenzene-1,4-diamine in the presence of acetic acid and acetonitrile as solvent.

## Graphical abstract

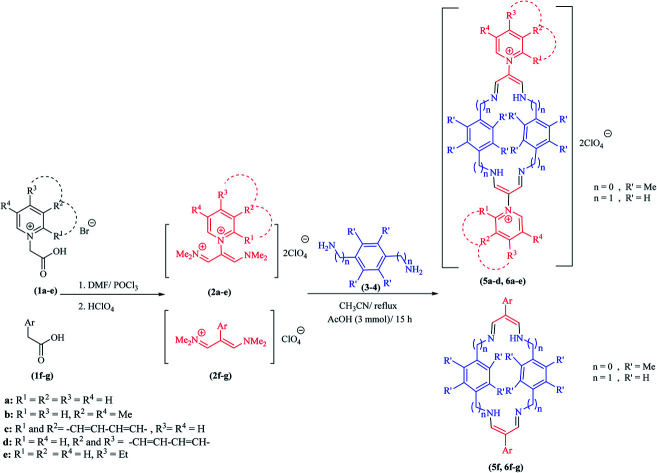

## Introduction

The structure of a vast range of macromolecules^1^ confirmed that the design and construction of macrocyclic compounds has been one of the most important reasons for improvement in supramolecular science. A well-known group of macrocyclic compounds are cyclophanes and their particular chemistry has attracted the attention of researchers, recently^2^ and has been broadly discussed within the field of modern supra-molecular chemistry.^3,4^ Cyclophanes^5–42^ are constrained organic molecules consisting of aromatic ring(s) as well as aliphatic unit(s). The aromatic rings support the rigidity of their structure, while the aliphatic unit(s) create bridge(s) between the aromatic rings and cause the flexibility of the whole structure.

**Fig. 1:**
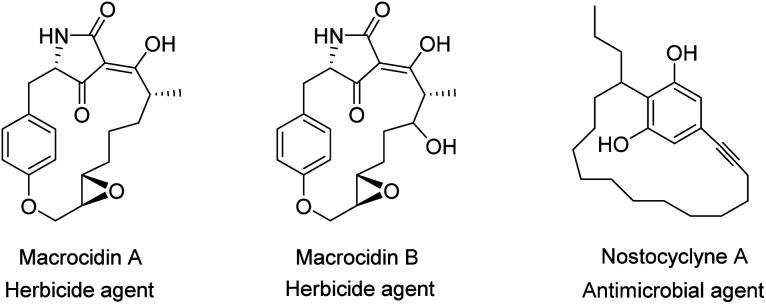
Natural products containing the cyclophane skeleton.

Due to their special structure, they are considered as an important class of compounds in “host–guest” chemistry^43–47^ and supramolecular assembly.^48–51^ On the other hand, the clear structure and high strain of cyclophanes has contributed in a large number of applications such as pharmaceuticals,^52,53^ asymmetric catalysis,^54^ insulating plastics,^55^ organic electronics,^56^ metal capture^57,58^ and supramolecular chemistry.^59^ Besides, since cyclophane structure is the main foundation unit in many biologically active natural products (Fig. 1),^60–62^ their design and application is a special interest of groups working in fields including biological, medicinal and organic chemistry. Moreover, cyclophanes are attractive in the area of theoretical chemistry due to their particular topology and intra-molecular interactions.

**Scheme 1:**
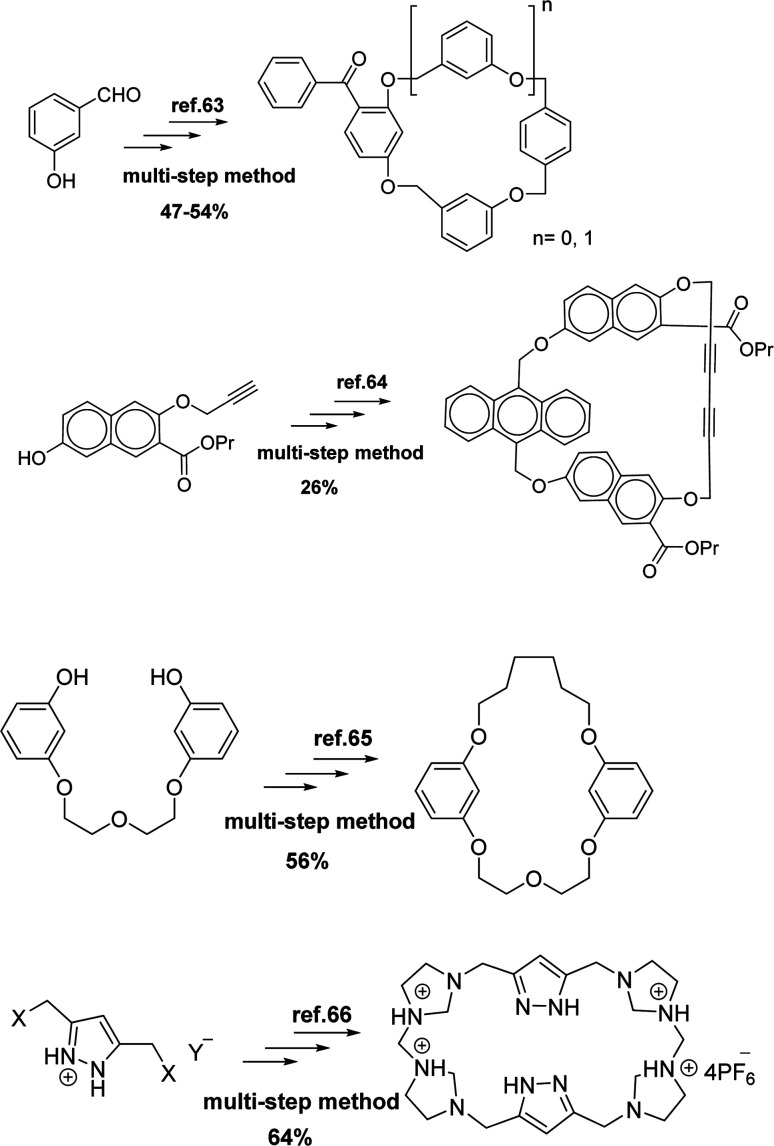
Reported methods for the synthesis of cyclophanes.

So far, several methods have been reported for the synthesis of cyclophanes compounds^63–66^ (Scheme 1). In some instances, cyclophanes are formed in low yields and also with side products. Some procedures require many steps or harsh reaction conditions such as high-pressure, and difficult separation methods. Therefore, designing better routes and improving conditions to achieve cyclophanes is still needed.

**Scheme 2:**
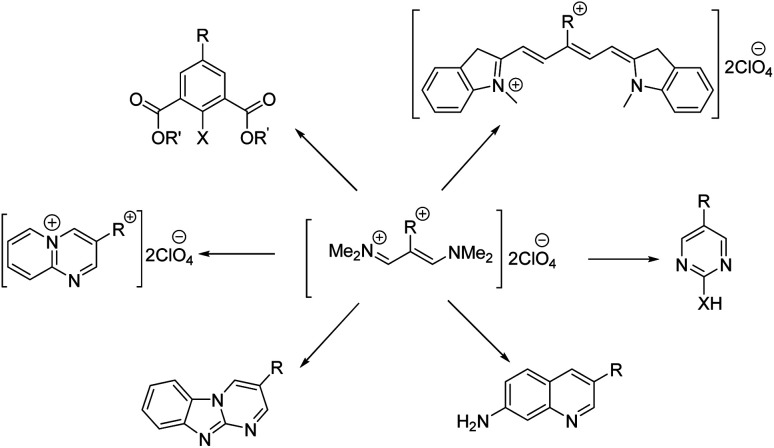
Application of vinamidinium salts in organic synthetic chemistry.

Vinamidinium salts are examples of stabilized alkenes by “push–pull” influences between the electron-donating amino group and the electron-withdrawing ammonium group. They can easily undergo condensation reaction with bifunctional nucleophiles to form heterocycles. During past years, our group has been investigated the utilization of vinamidinium salts for the synthesis of heterocyclic compounds^67–76^ (Scheme 2).

## Results and discussion

**Scheme 3:**
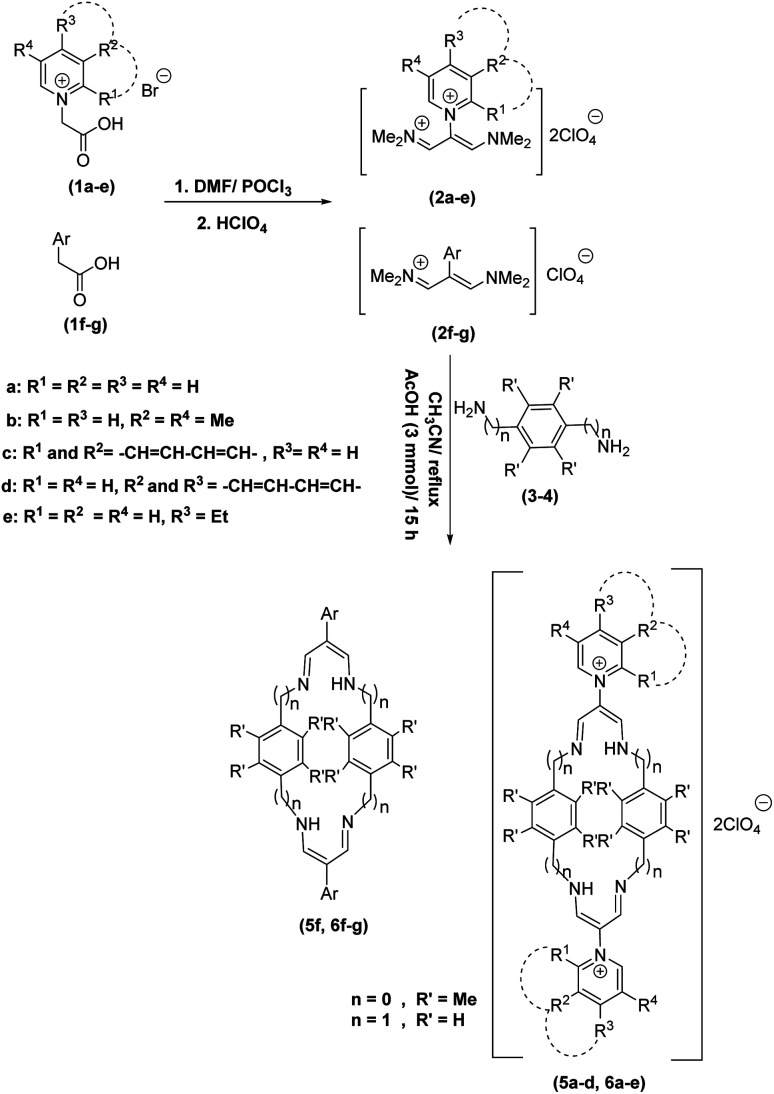
Synthesis of cyclophanes.

In continuation of these studies and due to the importance of cyclophanes in different branches of sciences, we decided to investigate the one-pot synthesis of cyclophanes from vinamidinium salts (Scheme 3). To the best of our knowledge, vinamidinium salts have not yet been applied for preparing cyclophanes.

As shown in Scheme 3, the procedure is done in two-step: (i) synthesis of the 2-substituted vinamidinium salts (2a–g) by the Vilsmeier–Arnold formylation of the substituted acetic acids (1a–g) as explained in authors previous work;^67–76^ and (ii) synthesis of cyclophane derivatives (5a–f, 6a–g) by the reaction of 2-substituted vinamidinium salts (2a–g) with 1,4-phenylenedimethanamine (3) or 2,3,5,6-tetramethylbenzene-1,4-diamine (4) by applying acetic acid in acetonitrile as solvent.

In the first step, vinamidiniums were isolated as the perchlorate salts and used directly without additional purification for the synthesis of cyclophanes.

As illustrated in Scheme 3, the symmetrical vinamidinium salts (2a–g) were able to react with 1,4-phenylenedimethanamine (3) or 2,3,5,6-tetramethylbenzene-1,4-diamine (4) in refluxing acetonitrile in the presence of acetic acid for 15 h to manage the cyclophane derivatives (5a–f, 6a–g). To provide the best reaction conditions in second step, the reaction of vinamidinium salt 2f with 1,4-phenylenedimethanamine (3) was chosen as model reaction and the impacts of solvents and catalysts were investigated. The obtained results are summarized in Table 1. When EtOH and MeOH were applied as the solvent and the mixture was subjected to reflux in the presence of AcOH, the desired product, 5f, was achieved in low yields (43% and 36%, respectively) after 24 h (Table 1, entries 1 and 2). In a modified protocol, the reaction was carried out in refluxing acetonitrile. In this case, remarkable improvement of yield was observed (Table 1, entry 3). No better results were obtained when the reaction was carried out in DMF, CHCl_3_, CH_2_Cl_2_ or toluene (Table 1, entries 4–7). Therefore, the subsequent reactions were carried out in CH_3_CN.

**Table 1:** Effect of the different reaction parameters on the reaction of 1,4-phenylenedimethanamine (3) with ((*E*)-*N*-(3-(dimethylamino)-2-(naphthalen-1-yl)allylidene)-*N*-methylmethanaminium perchlorate (2f))

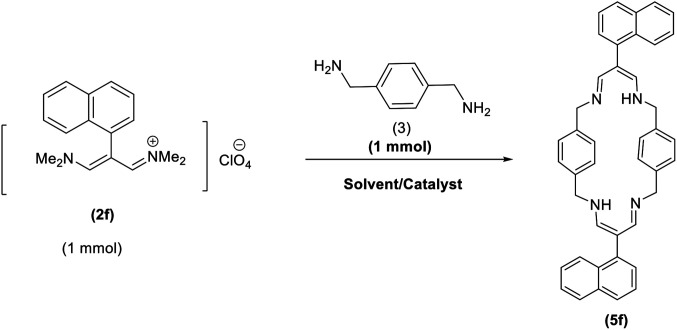				
Entry	Condition	Solvent	Time (h)	Yield^a^ (%)
1	AcOH (3 mmol)	EtOH/reflux	24	43
2	AcOH (3 mmol)	MeOH/reflux	24	36
3	AcOH (3 mmol)	CH_3_CN/reflux	15	90
4	AcOH (3 mmol)	CHCl_3_/reflux	24	35
5	AcOH (3 mmol)	CH_2_Cl_2_/reflux	24	Trace
6	AcOH (3 mmol)	Toluene/reflux	24	—
7	AcOH (3 mmol)	DMF/100 °C	24	45
8	Et_3_N (3 mmol)	CH_3_CN/reflux	24	—
9	i-Pr_2_NEt (3 mmol)	CH_3_CN/reflux	24	—
10	—	CH_3_CN/reflux	24	—
11	AcOH (4 mmol)	CH_3_CN/reflux	15	90
12	AcOH (2 mmol)	CH_3_CN/reflux	24	64
13	AcOH (1 mmol)	CH_3_CN/reflux	24	40

a Isolated yield.

After choosing the solvent, the model reaction was conducted under neutral and basic conditions. As shown in entries 8 and 9 of Table 1, in the presence of basic catalysts such as triethylamine and ethyldiisopropylamine, the desired product 5f, was not achieved. In neutral conditions also, no product was formed (Table 1, entry 10). Increasing the amount of AcOH did not affect the reaction appreciably, while, decreasing the amount of AcOH, resulted the product in lower yield (Table 1, entries 11–13). So, acidic media is critical to the success of the reaction.

We then applied the obtained optimized conditions for the reaction of different vinamidinium salts with 1,4-phenylenedimethanamine (3). The results are listed in Table 2.

**Table 2:** The synthesis of cyclophane derivatives from the reaction of 2-substituted vinamidinium salts (2a–g) (1.0 mmol), 1,4-phenylenedimethanamine (3) (1.0 mmol) in the presence of AcOH (3.0 mmol) in CH_3_CN (8.0 mL) at reflux conditions

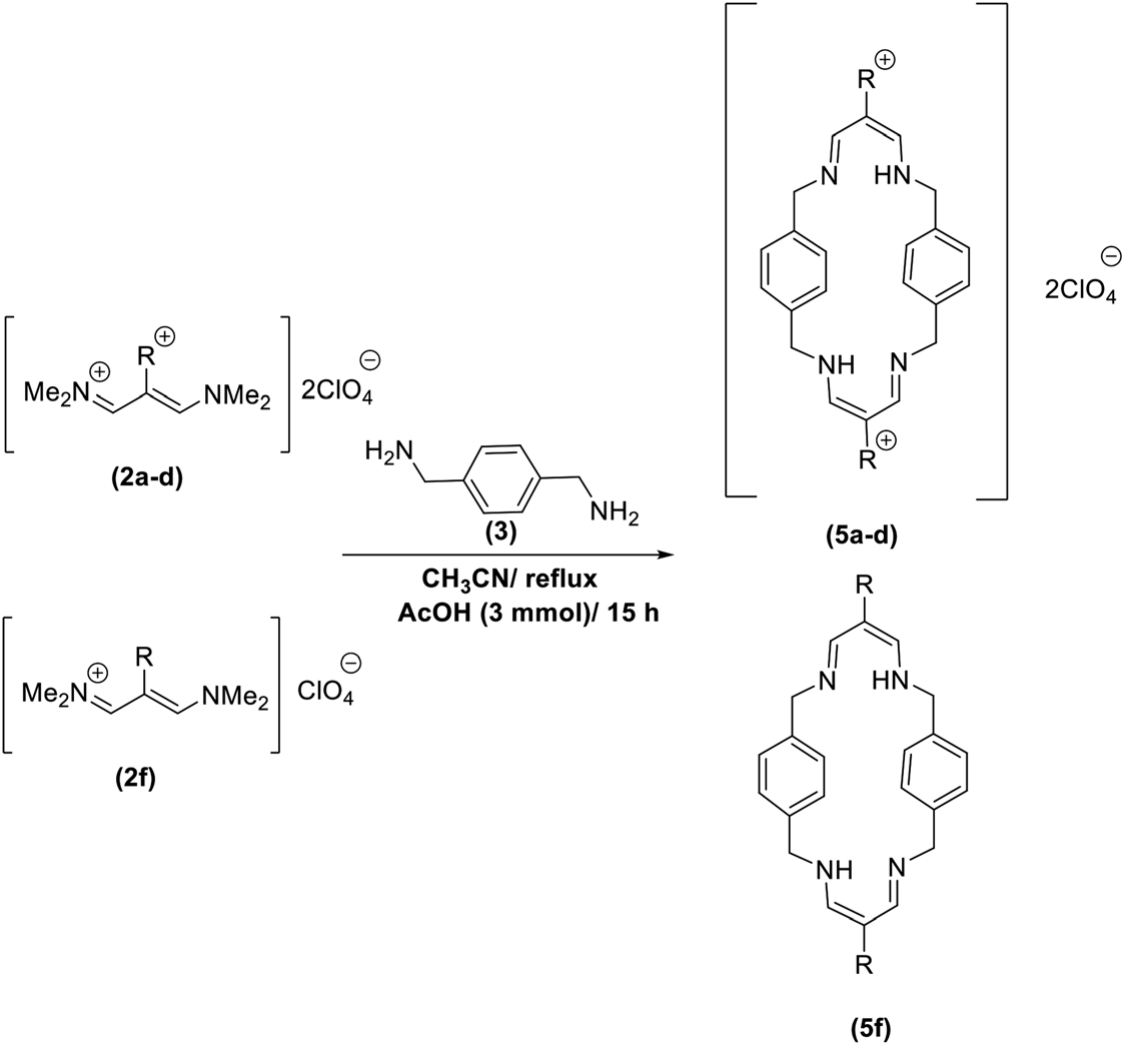		
Entry	R^+^ or R	Yield^a^ (%)
1	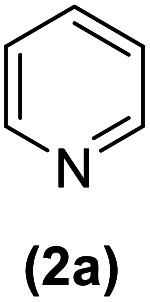	87
2	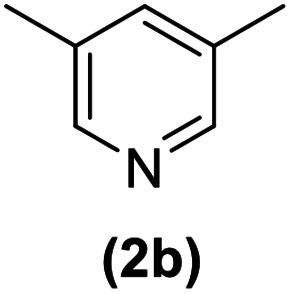	90
3	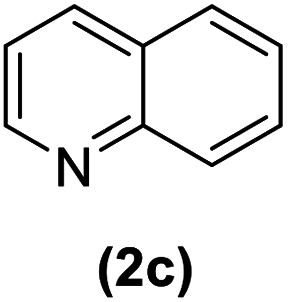	83
4	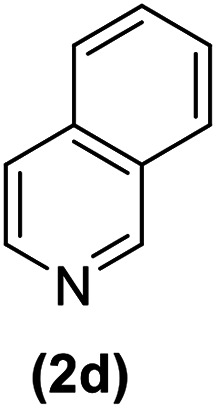	88
5	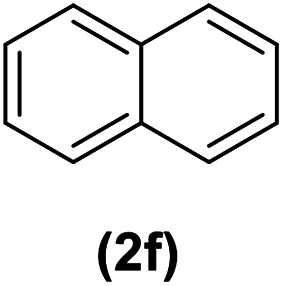	90

a Isolated yield.

The results demonstrated that the variety of vinamidinium salts were successfully employed in this process, affording novel cyclophanes in high yields in suitable reaction times.

After successful application of 1,4-phenylenedimethanamine (3) in cyclophane synthesis, to enhance the generality of the system, we applied 2,3,5,6-tetramethylbenzene-1,4-diamine (4) as bifunctional nucleophile and investigated the formation of new cyclophanes.

2,3,5,6-Tetramethylbenzene-1,4-diamine (4) was also tolerated well in this procedure to give the desired products (Table 3).

**Table 3:** The synthesis of cyclophane derivatives from the reaction of 2-substituted vinamidinium salts (2a–g) (1.0 mmol), 2,3,5,6-tetramethylbenzene-1,4-diamine (4) (1.0 mmol) in the presence of AcOH (3.0 mmol) in CH_3_CN (8.0 mL) at reflux conditions

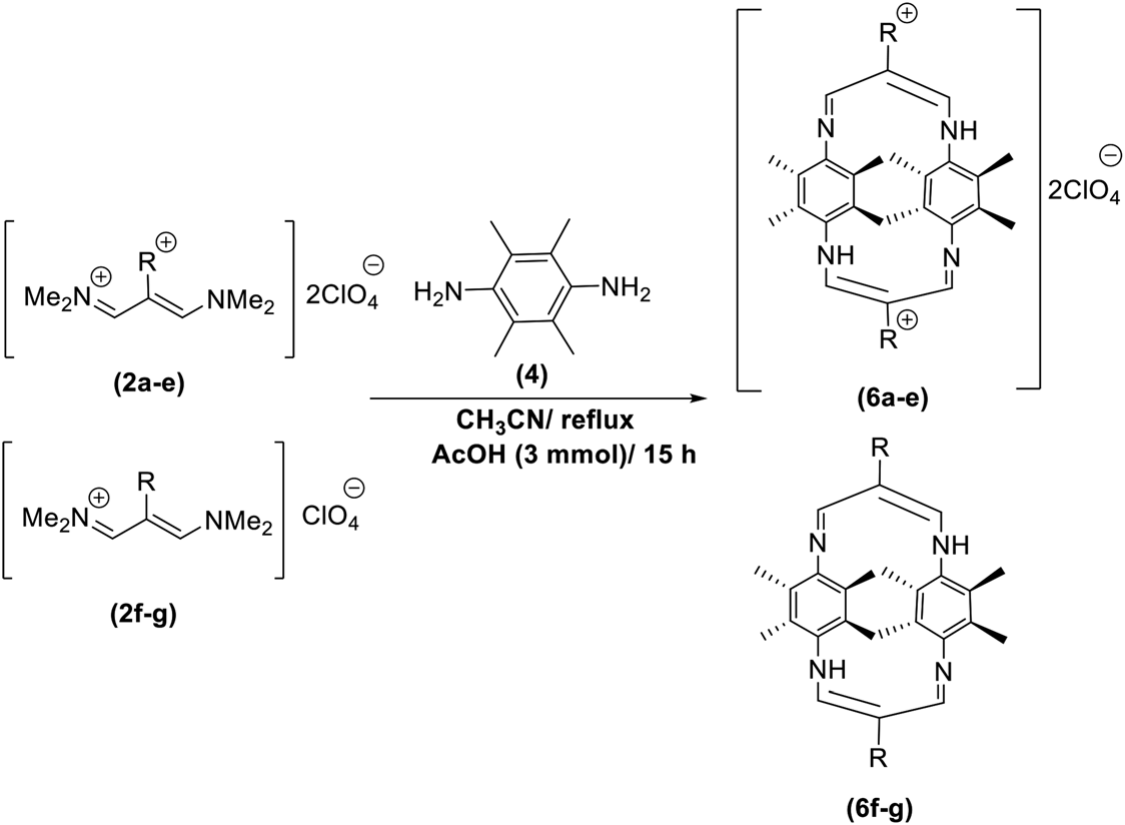		
Entry	R^+^ or R	Yield^a^ (%)
1	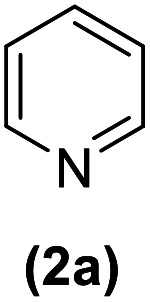	77
2	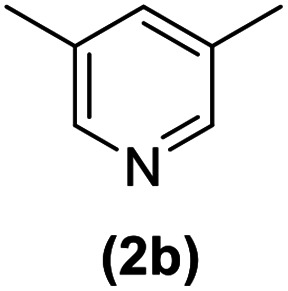	86
3	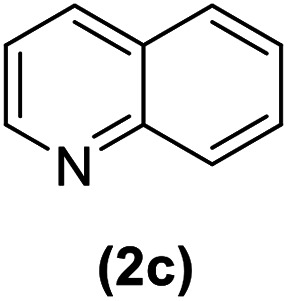	80
4	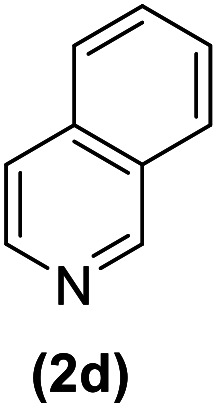	82
5	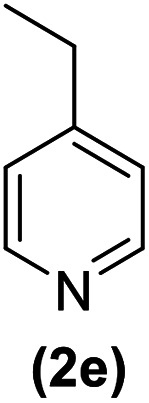	81
6	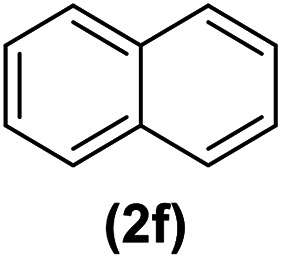	90
7	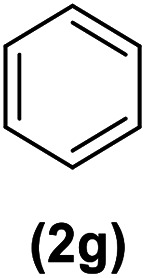	87

a Isolated yield.

**Scheme 4:**
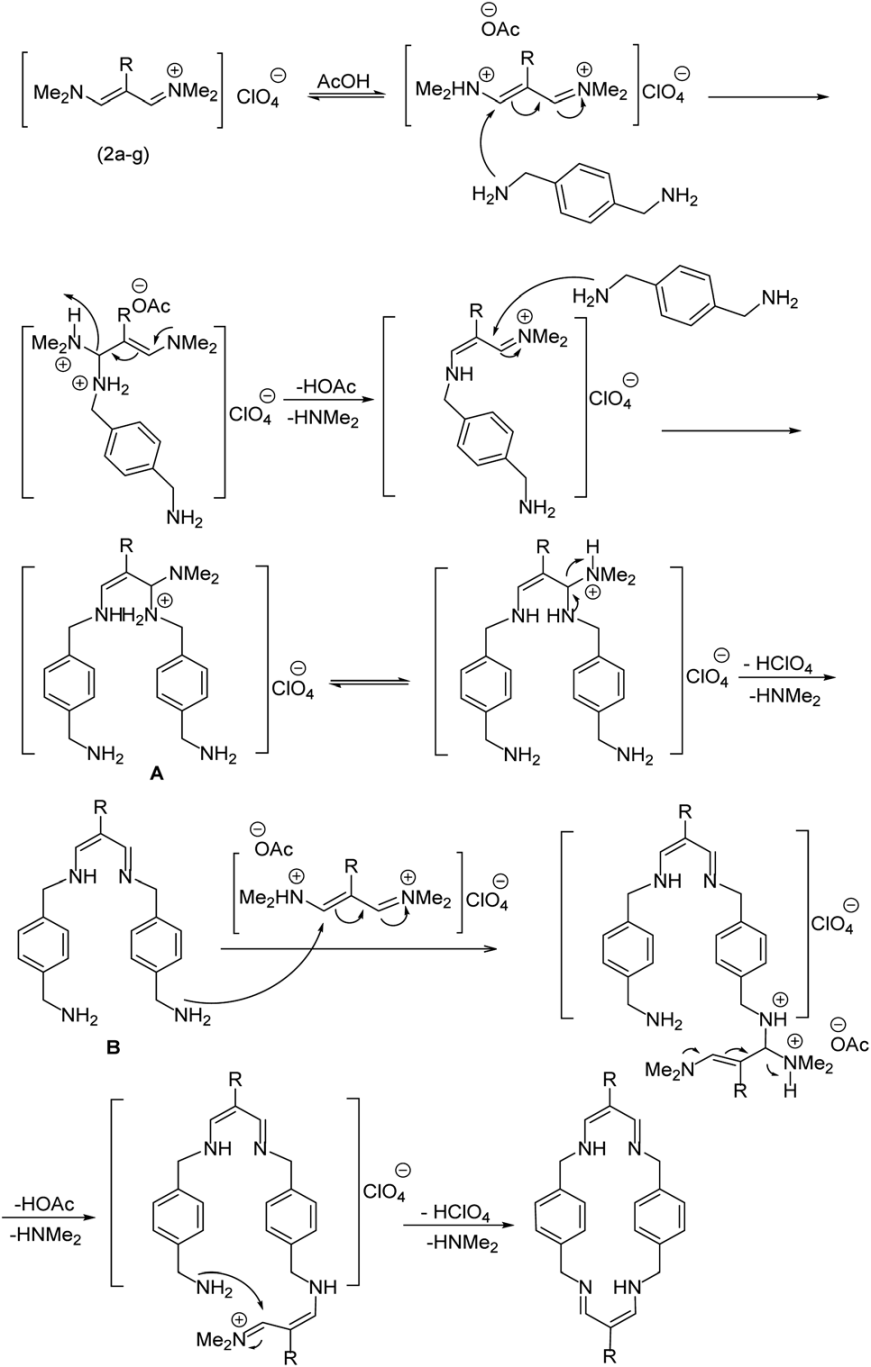
The proposed mechanism for the synthesis of cyclophanes in the presence of AcOH.

According to our previous works and above results, a reasonable mechanism for the synthesis of cyclophane compounds (5a–f, 6a–g) in the presence of AcOH is illustrated in Scheme 4.

The reaction proposed by the initial attack of the amino group in 1,4-phenylenedimethanamine (3) or 2,3,5,6-tetramethylbenzene-1,4-diamine (4) to protonated vinamidinium salt. Then, removal of dimethylamine occurs, followed by the nucleophilic attack of second molecule of amine on the obtained iminium salt to produce intermediate A. The loss of the second dimethylamine molecule produces intermediate B. The reaction of this intermediate with the second molecule of vinamidinium salt, followed by the loss of two dimethylamine molecules and intramolecular nucleophilic cyclization, yields the desired cyclophane.

## Conclusion

In this study, an efficient and applicable protocol has been developed for the synthesis of novel cyclophanes by the reaction of 2-substituted vinamidinium salts with 1,4-phenylenedimethanamine or 2,3,5,6-tetramethylbenzene-1,4-diamine in the presence of acetic acid.

This protocol has several advantages such as: simple and one-step procedure, absence of by-products, inexpensive catalyst, normal atmospheric conditions, high to excellent yields and easy purification of the products. Furthermore, the products are well-known, stable solids and have a long shelf-life when stored in an anhydrous environment.

## Experimental section

### General procedure for the synthesis of cyclophane derivatives (5a–f, 6a–g)

To a flame dried one-necked round-bottomed flask equipped with magnetic stirring and reflux condenser, 2-substituted vinamidinium salts (2a–g) (1.0 mmol), 1,4-phenylenedimethanamine (3) or 2,3,5,6-tetramethylbenzene-1,4-diamine (4) (1.0 mmol), AcOH (3.0 mmol) and CH_3_CN (8.0 mL) were added. The mixture was allowed to reflux for 15 h in an oil bath. After completion of the reaction, distilled H_2_O (20 mL) was added to the mixture. The resulting precipitate was gathered, washed with Et_2_O (3 × 3 mL). Finally, the precipitate was washed with 2-propanol (3 × 3 mL) and dried under vacuum at 80 °C to afford the corresponding cyclophanes.

## Conflicts of interest

There are no conflicts of interest to declare.

## Supplementary Material

RA-011-D0RA10548A-s001
