# Supplementary material for: A facile and efficient route to one-pot synthesis of new cyclophanes using vinamidinium salts
Source: RSC Adv. 2021 Apr 13;11(22):13666–73. doi: 10.1039/d0ra10548a (PMC8697689; doi:10.1039/d0ra10548a)
Supplement: RA-011-D0RA10548A-s001 [file RA-011-D0RA10548A-s001.pdf]

Electronic Supplementary Material (ESI) for RSC Advances.
This journal is © The Royal Society of Chemistry 2021

Supporting Information

A facile and efficient route to one-pot synthesis of new cyclophanes using vinamidinium salts

Nooshin Golzar,[a] Abdolmohammad Mehranpour [a]* and Najmeh Nowrouzi [a]

[a]*Department of Chemistry, Faculty of Sciences, Persian Gulf University, Bushehr 75169, Iran.*

Corresponding author. Tel.: +98-77-31223357; Fax: +98-77-33441494.

ammehranpour@hotmail.com.

List of contents

1. General information……………………………………………………………………2

2. Synthesis of cyclophane derivatives………..……………………………………….....…..2
General procedure

3. Analytical data.....................…………………………………………….……….....…....2

4. Spectra............……………………………………………………………..……......…..9

1. General information

Commercially available reagents were used without further purification. All reactions were carried out under atmospheric pressure in a closed vessel and progress of reactions were monitored by TLC analysis. Purification of all products were performed by column chromatography using n-hexane/ethyl acetate as eluent. NMR spectra were recorded at room temperature in DMSO on a 400 MHz NMR spectrometer, with tetramethylsilane (TMS) as internal standard. Chemical shifts (δ) are reported in ppm and coupling constants are listed in hertz. Multiplicity is reported as follows: s = singlet, d = doublet, t = triplet, q = quartet, m = multiplet, dd = doublet of doublet, dt = doublet of triplet, tt = triplet of triplet.

2. Synthesis of cyclophane derivatives (5a-f, 6a-g)

General procedure (All derivatives were synthesized trough the same procedure. Here is an example for the formation of compound **5d**): To a flame dried one-necked round-bottomed flask equipped with magnetic stirring and reflux condenser, 2-(1-(dimethylamino)-3-(dimethyliminio)prop-1-en-2-yl)isoquinolin-2-ium bis(perchlorate)(**2d)** (1.0 mmol: 432.0mg), 1,4-phenylenedimethanamine (**3**) (1.0 mmol: 136.0 mg), AcOH (3.0 mmol: 0.17mL) and CH_3CN (8.0 mL) as solvent were added. The mixture was allowed to reflux for 15 h in an oil bath. After completion of the reaction, distilled H_2O (20 mL) was added to the mixture. The resulting precipitate was gathered, washed with Et_2O (3 × 3 mL). Finally, the precipitate was washed with 2-propanol (3 × 3 mL) and dried under vacuum at 80°C to afford the corresponding cyclophanes.

3. Analytical data

3,7,11,15-Tetraaza-1,9(1,4) -dibenzenacyclohexadecaphane-3,5,11,13-tetraene-5,13-diyl)bis(pyridin-1-ium)-bis(perchlorate) (5a)

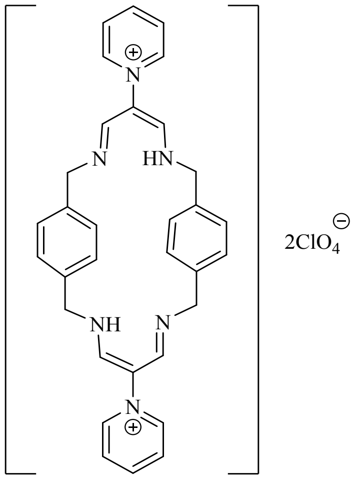

(5a)

Dark brown powder; Yield (87%); mp > 250 °C; IR (KBr), ῡ (cm^{-1}): 3414, 1651, 1095; ^{1}H NMR (400 MHz, DMSO-d_6) δ (ppm): 4.49 (s, 8H, CH_2), 7.36-7.52 (m, 6H, Ar), 8.28-8.40 (m, 2H, Ar), 8.52 (m, 5H, Ar), 8.72-8.86 (m, 2H, Ar), 8.94 (d, 2H, *J* = 8.0 Hz, Ar), 9.05 (d, 5H, *J* = 4.0 Hz, Ar), 10.25-10.29 (broad peak, 2H, NH). ^{13}C-NMR (100 MHz, DMSO-d_6) δ (ppm): 50.7, 111.8, 128.2, 138.2, 138.5, 142.1, 147.0, 156.1; EI-MS (70ev): m/z = 500[M^+-$2ClO_4^-$]; Anal. calcd for $(C_{32}H_{32}N_6)(CClO_4)_2$: C, 54.94; H, 4.61; N, 12.01. Found: C, 54.89; H, 4.58; N, 11.97.

3,7,11,15-Tetraaza-1,9(1,4)-dibenzenacyclohexadecaphane-3,5,11,13-tetraene-5,13-diyl)bis(3,5-dimethylpyridin-1-ium)-bis(perchlorate) (5b)

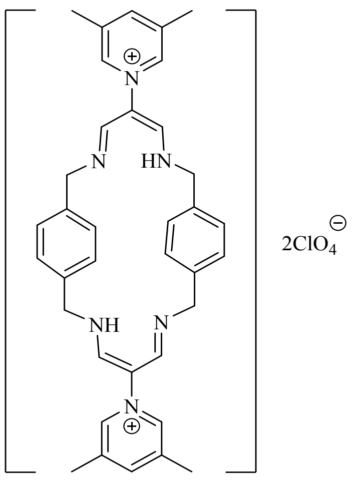

(5b) Orange powder; Yield (90%); mp > 250 °C; IR (KBr), ῡ (cm^{-1}): 3414, 1635, 1090 ; ^{1}H NMR (400 MHz, DMSO-d_6) δ (ppm): 3.37 (s, 12H, CH_3), 4.60 (s, 8H, CH_2), 8.14 (d, 7H, J = 6.0 Hz, Ar), 8.26 (s, 4H, Ar), 8.84 (s, 7H, Ar), 10.66 (s, 2H, NH). ^{13}C-NMR (100 MHz, DMSO-d_6) δ (ppm): 18.1, 57.8, 113.8, 128.2, 138.2, 138.5, 141.5, 144.9, 153.0; EI-MS (70ev): m/z = 556[M^+-2ClO_4^-]; Anal. calcd for ($C_{38}H_{40}N_6$) $(CClO_4)_2$: C, 58.54; H, 5.17; N, 10.17. Found: C, 58.49; H, 5.10; N, 10.69.

3,7,11,15-Tetraaza-1,9(1,4)-dibenzenacyclohexadecaphane-3,5,11,13-tetraene-5,13-diyl)bis(quinolin-1-ium)-bis(perchlorate) (5c)

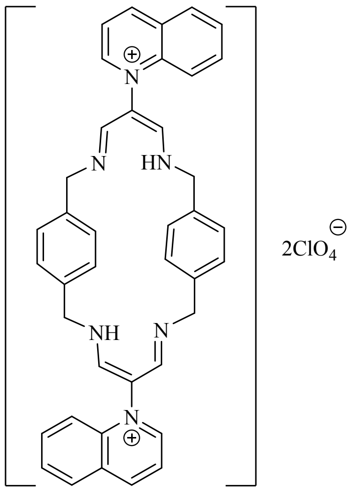

(5c) Dark brown powder; Yield (83%); mp > 250 °C; IR (KBr), ῡ (cm^{-1}): 3414, 1654, 1027; ^{1}H NMR (400 MHz, DMSO-d_6) δ (ppm): 4.06 (s, 8H, CH_2), 7.31-7.42 (m, 6H, Ar), 7.49-7.60 (m, 8H, Ar), 7.68 (d, 2H, J = 7.2 Hz, Ar), 7.88-7.98 (m, 5H, Ar), 8.07-8.21 (m, 5H, Ar), 10.05-10.12 (broad peak, 2H, NH). ^{13}C-NMR (100 MHz, DMSO-d_6) δ (ppm): 51.7, 111.2, 114.1, 116.9, 119.8, 128.3, 129.5, 129.8, 130.0, 148.3, 158.3, 158.7, 159.1, 159.4; EI-MS (70ev): m/z = 600[M^+-2ClO_4^-]; Anal. calcd for ($C_{42}H_{36}N_6$) $(CClO_4)_2$: C, 61.24; H, 4.41; N, 10.20. Found: C, 61.02; H, 4.39; N, 10.03.

3,7,11,15-Tetraaza-1,9(1,4)-dibenzenacyclohexadecaphane-3,5,11,13-tetraene-5,13-diyl)bis(isoquinolin-2-ium)-bis(perchlorate) (5d)

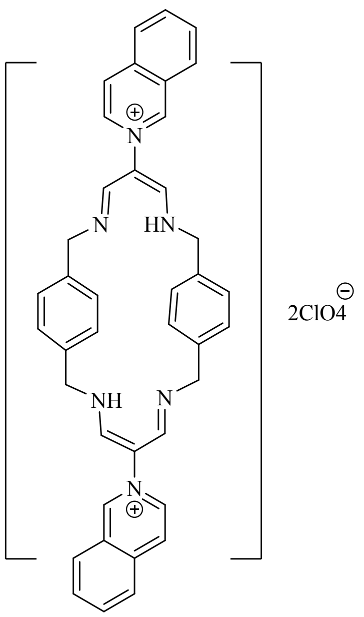

(5d) Yellow powder; Yield (88%); mp > 250 °C; IR (KBr), ῡ (cm^{-1}): 3416, 1601, 1089; ^{1}H NMR (400 MHz, DMSO-d_6) δ (ppm): 4.53 (s, 8H, CH_2), 7.31-7.35 (m, 4H, Ar), 7.55 (s, 8H, Ar), 8.27 (t, 2H, J = 8.0 Hz, Ar), 8.52 (d, 2H, J = 8.0 Hz, Ar), 8.59 (d, 2H, J = 8.0 Hz, Ar), 8.66 (d, 4H, J = 8.0 Hz, Ar), 8.98 (d, 2H, J = 4.0 Hz, Ar), 10.10 (s, 4H, Ar), 10.13 (s, 2H, NH). ^{13}C-NMR (100 MHz, DMSO-d_6) δ (ppm): 50.9, 111.9, 128.1, 129.2, 130.0, 131.5, 132.1, 133.4, 137.8, 138.9, 139.1, 155.3, 155.7, 159.4; EI-MS (70ev): m/z = 600[M^{+}-2ClO_4^{-}]; Anal. calcd for ($C_{42}H_{36}N_6$) ($CClO_4$)$_2$: C, 61.24; H, 4.41; N, 10.20. Found: C, 61.00; H, 4.22; N, 10.15.

5,13-Di(naphthalen-1-yl)-3,7,11,15-tetraaza-1,9(1,4) dibenzenacyclohexadecaphane-4,6,12,14-tetraene (5f)

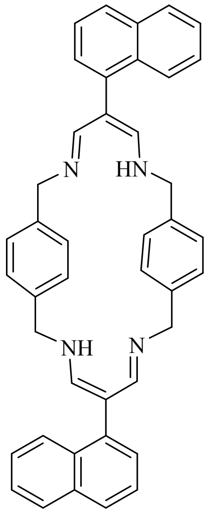

(5f) White powder; Yield (90%); mp > 250 °C; IR (KBr), ῡ (cm^{-1}): 3415, 1629; ^{1}H NMR (400 MHz, DMSO-d_6) δ (ppm): 4.09 (s, 8H, CH_2), 7.48-7.50 (m, 4H, Ar), 7.58-7.71 (m, 8H, Ar), 7.81 (d, 2H, J = 8.0 Hz, Ar), 7.97 (m, 6H, Ar), 8.06 (t, 6H, J = 6.0 Hz, Ar), 8.14 (s, 2H, NH). ^{13}C-NMR (100 MHz, DMSO-d_6) δ (ppm): 49.0, 102.2, 125.7, 126.1, 127.2, 128.1, 129.1, 129.5, 129.9, 130.3, 130.9, 133.2, 134.1 , 134.6, 164.3; EI-MS (70ev): m/z = 596[M^{+}]; Anal. calcd for $C_{42}H_{36}N_4$: C, 84.53; H, 6.08; N, 9.39. Found: C, 85.04; H, 5.89; N, 9.51.

Octamethyl-2,6,8,12-tetraaza-1,7(1,4)-dibenzenacyclododecaphane-3,5,9,11-tetraene-4,10-diyl)bis(pyridin-1-ium)- bis(perchlorate) (6a)

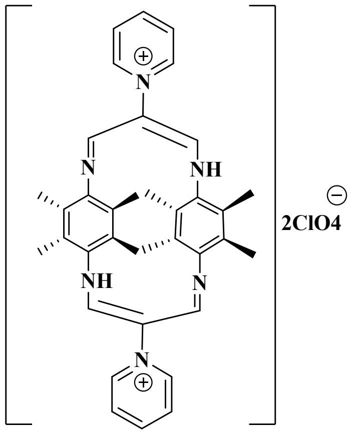

(6a)

Red powder; Yield (77%); mp > 250 °C; IR (KBr), ῡ (cm^{-1}): 3420, 1616, 1091 ; ^{1}H NMR (400 MHz, DMSO-d_6) δ (ppm): 2.47 (s, 24H, CH_3), 8.27 (t, 4H, J = 8.6 Hz, Ar), 8.59 (d, 4H, J = 7.6 Hz, Ar), 8.70 (t, 2H, J = 10.0 Hz, Ar), 9.32 (d, 4H, J = 7.2 Hz, Ar), 14.02 (s, 2H, NH). ^{13}C-NMR (100 MHz, DMSO-d_6) δ (ppm): 38.8, 49.9, 116.0, 120.1, 126.7, 128.3, 136.6, 145.6, 146.0, 147.9; EI-MS (70ev): m/z = 540[M^+-$2ClO_4^-$]; Anal. calcd for ($C_{37}H_{36}N_6$) $(CClO_4)_2$: C, 58.20; H, 4.75; N, 11.01. Found: C, 58.00; H, 4.69; N, 10.93.

Octamethyl-2,6,8,12-tetraaza-1,7(1,4)-dibenzenacyclododecaphane-3,5,9,11-tetraene-4,10-diyl)bis(3,5-dimethylpyridin-1-ium)- bis(perchlorate) (6b):

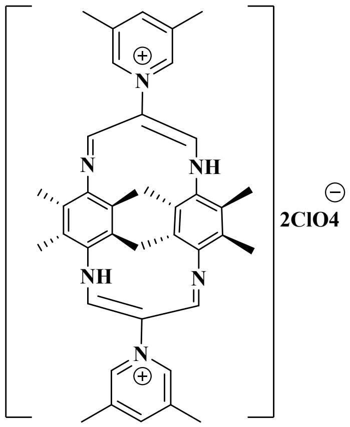

(6b)

Red powder; Yield (86%); mp > 250 °C; IR (KBr), ῡ (cm^{-1}): 3416, 1617, 1089 ; ^{1}H NMR (400 MHz, DMSO-d_6) δ (ppm): 2.55 (s, 24H, CH_3), 2.89 (s, 12H, CH_3), 8.41 (s, 2H, Ar), 8.57 (m, 4H, Ar), 9.05 (s, 4H, Ar), 13.99-14.02 (broad peak, 2H, NH). ^{13}C-NMR (100 MHz, DMSO-d_6) δ (ppm): 18.2, 38.7, 50.3, 116.1, 119.9, 126.7, 136.6, 138.2, 142.7, 146.3, 147.7 EI-MS (70ev): m/z = 596[M^+-$2ClO_4^-$];; Anal. calcd ($C_{39}H_{44}N_6$) $(CClO_4)_2$: C, 58.87; H, 5.57; N, 10.56. Found: C, 58.96; 5.42; N, 10.68.

Octamethyl-2,6,8,12-tetraaza-1,7(1,4)-dibenzenacyclododecaphane-3,5,9,11-tetraene-4,10-diyl)bis(quinolin-1-ium)-bis(perchlorate) (6c)

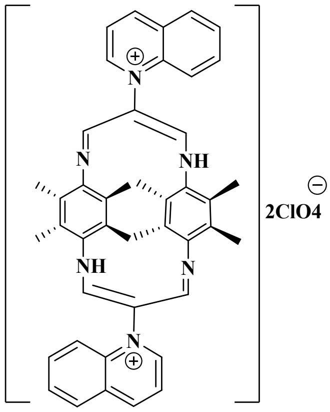

(6c) Dark brown powder; Yield (80%); mp > 250 °C; IR (KBr), ʋ (cm^{-1}): 3421, 1621, 1111; ^{1}H NMR (400 MHz, DMSO-d_6) δ (ppm): 2.17 (s, 24H, CH_3), 7.06 (s, 2H, Ar), 7.32 (t, 2H, J = 4.0 Hz, Ar), 8.15 (t, 2H, J = 6.6 Hz, Ar), 8.30-8.39 (m, 3H, Ar), 8.49-8.63 (m, 5H, Ar), 9.50 (d, 2H, J = 8.0 Hz, Ar), 9.74 (d, 2H, J = 4.2 Hz, Ar), 14.23-14.25 (m, 2H, (broad NH)). ^{13}C-NMR (100 MHz, DMSO-d_6) δ (ppm): 43.5, 55.3, 115.5, 116.0, 120.6, 122.8, 126.6, 130.2, 130.7, 136.6, 136.7, 141.3, 148.4,148.9, 153.1, 155.0; EI-MS (70ev): m/z = 640[M^+-2ClO_4^-]; Anal. calcd for calcd ($C_{43}H_{40}N_6$) $(CClO_4)_2$: C, 61.50; H, 4.80; N, 10.01. Found: C, 61.37; H, 4.68; N, 10.16.

Octamethyl-2,6,8,12-tetraaza-1,7(1,4)-dibenzenacyclododecaphane-3,5,9,11-tetraene-4,10-diyl)bis(isoquinolin-2-ium)-bis(perchlorate) (6d)

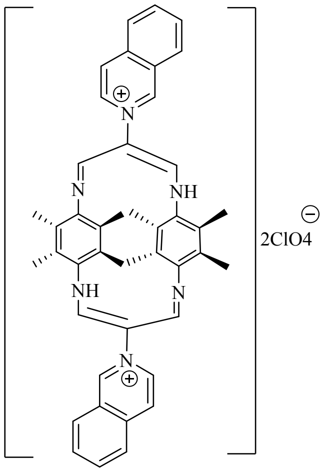

(6d) Red powder; Yield (82%); mp = 248-250 °C; IR (KBr), ʋ (cm^{-1}): 3417, 1628, 1093; ^{1}H NMR (400 MHz, DMSO-d_6) δ (ppm): 2.18 (s, 24H, CH_3), 7.19 (s, 2H, Ar), 7.56 (s, 2H, Ar), 8.18-8.36 (m, 2H, Ar), 8.45-8.57 (m, 3H, Ar), 8.74 (m, 5H, Ar), 9.08 (s, 2H, Ar), 10.34 (s, 2H, Ar), 14.13 (s, 2H, NH). ^{13}C-NMR (100 MHz, DMSO-d_6) δ (ppm): 38.4, 50.2, 115.9, 120.1, 125.9, 126.9, 127.6, 127.8, 131.1, 131.8, 136.5, 136.7, 137.1, 137.6, 147.8, 150.6; EI-MS (70ev): m/z = 640[M^+-2ClO_4^-]; Anal. calcd for ($C_{45}H_{40}N_6$) $(CClO_4)_2$: C, 62.57; H, 4.67; N, 9.37. Found: C, 62.47; H, 4.53; N, 9.17.

Octamethyl-2,6,8,12-tetraaza-1,7(1,4)-dibenzenacyclododecaphane-3,5,9,11-tetraene-4,10-diyl)bis(4-ethylpyridin-1-ium)-bis(perchlorate) (6e)

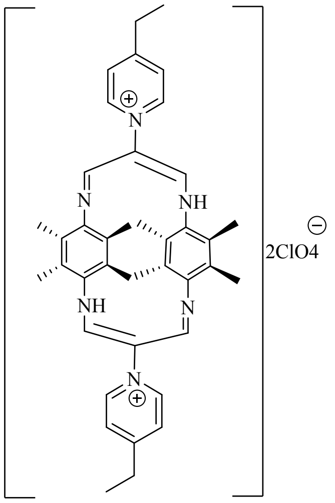

(6e) Dark red powder; Yield (81%); mp > 250 °C; IR (KBr), ῡ (cm^{-1}): 3413, 1633, 1088; ^{1}H NMR (400 MHz, DMSO-d_6) δ (ppm): 1.34 (t, 6H, J = 6.0 Hz, CH_3), 2.18 (s, 24H, CH_3), 2.83-3.02 (m, 4H, CH_2), 7.52-7.59 (m, 4H, Ar), 8.16 (d, 2H, J = 5.6 Hz, Ar), 8.57 (d, 2H, J = 6.4 Hz, Ar), 9.06-9.09 (m, 4H, Ar), 13.98-14.01 (broad peak, 2H, NH). ^{13}C-NMR (100 MHz, DMSO-d_6) δ (ppm): 14.3, 28.5, 38.8, 50.2, 115.9, 119.7, 127.5, 136.6, 138.4, 142.7, 147.6, 148.2; EI-MS (70ev): m/z = 596[M^+-2ClO_4^-]; Anal. calcd for ($C_{41}H_{44}N_6$) ($CClO_4$)$_2$: C, 60.07; H, 5.41; N, 10.25. Found: C, 60.00; H, 5.32; N, 10.30.

Octamethyl-4,10-di(naphthalen-1-yl)-2,6,8,12-tetraaza-1,7(1,4)dibenzenacyclododecaphane-3,5,9,11-tetraene (6f)

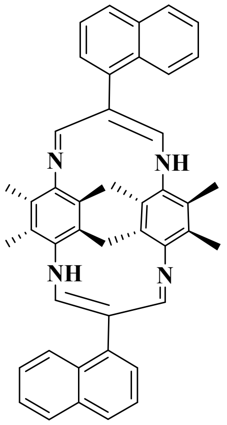

(6f) Red powder; Yield (90%); mp > 250 °C; IR (KBr), ῡ (cm^{-1}): 3415, 1617; ^{1}H NMR (400 MHz, DMSO-d_6) δ (ppm): 2.15 (s, 24H, CH_3), 7.47-7.49 (dd, 2H, J_1 = 6.8 Hz, J_2 = 1.2 Hz, Ar), 7.58-7.70 (m, 7H, Ar), 7.80 (d, 2H, J = 8.4 Hz, Ar), 7.96 (s, 4H, Ar), 8.06 (t, 3H, J = 6.6 Hz, Ar), 8.15 (s, 2H, NH). ^{13}C-NMR (100 MHz, DMSO-d_6) δ (ppm): 38.7, 49.0, 102.5, 125.7, 126.1, 127.1, 128.0, 129.1, 129.9, 130.3, 130.9, 133.3, 134.1, 154.0, 154.6, 164.4, 164.5; EI-MS (70ev): m/z = 636[M^+]; Anal. calcd for $C_{45}H_{40}N_4$: C, 84.87; H, 6.33; N, 8.80. Found: C, 84.69; H, 6.18; N, 8.61.

Octamethyl-4,10-diphenyl-2,6,8,12-tetraaza-1,7(1,4)-dibenzenacyclododecaphane-3,5,9,11-tetraene (6g)

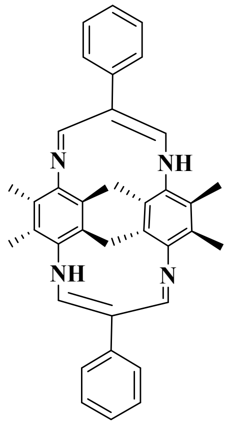

(6g) Pale pink powder; Yield (87%); mp > 250 °C; IR (KBr), ῡ (cm^{-1}): 3385, 1588; ^{1}H NMR (400 MHz, DMSO-d_6) δ (ppm): 2.43 (s, 24H, CH_3), 7.31-7.36 (m, 4H, Ar), 7.45 (t, 6H, J = 4.2 Hz, Ar), 7.72 (s, 4H, Ar), 9.39 (s, 2H, NH). ^{13}C-NMR (100 MHz, DMSO-d_6) δ (ppm): 38.6, 49.0, 105.5, 128.8, 129.2, 132.6, 133.0, 142.0, 142.6, 154.8, 163.3; EI-MS (70ev): m/z = 536[M^+]; Anal. calcd for $C_{37}H_{36}N_4$: C, 82.80; H, 6.76; N, 10.44. Found: C, 84.62; H, 6.62; N, 10.47.

4. Spectra

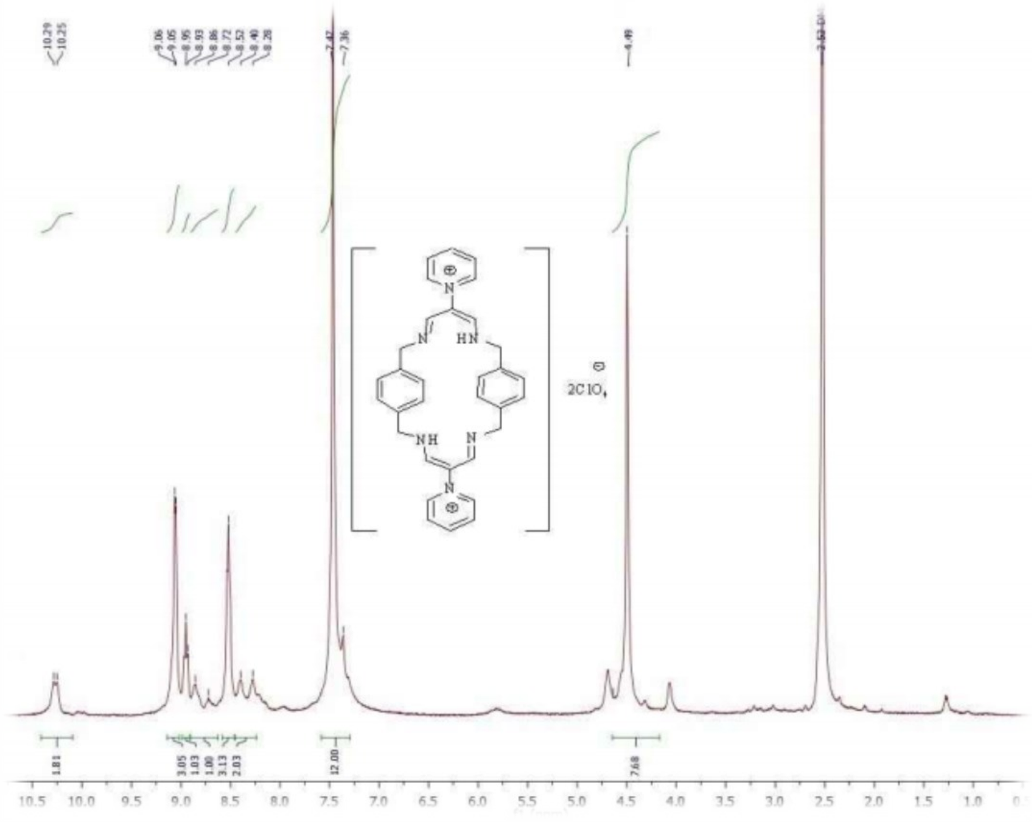

^{1}HNMR of 3,7,11,15-Tetraaza-1,9(1,4) -dibenzenacyclohexadecaphane-3,5,11,13-tetraene-5,13-diyl)bis(pyridin-1-ium)-bis(perchlorate) (5a)

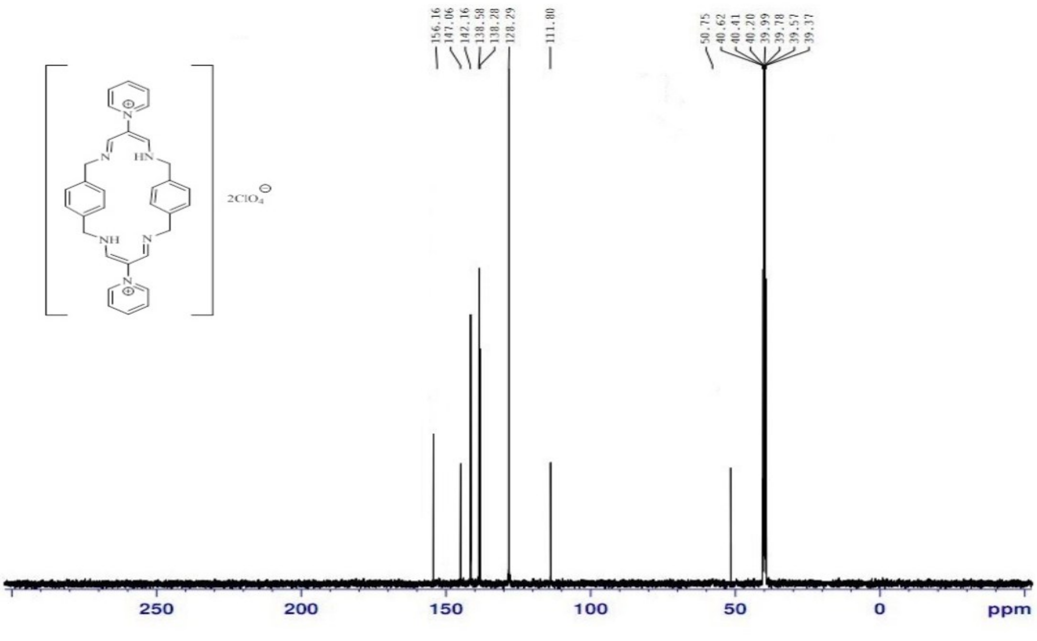

^{13}CNMR of 3,7,11,15-Tetraaza-1,9(1,4) -dibenzenacyclohexadecaphane-3,5,11,13-tetraene-5,13-diyl)bis(pyridin-1-ium)-bis(perchlorate) (5a)

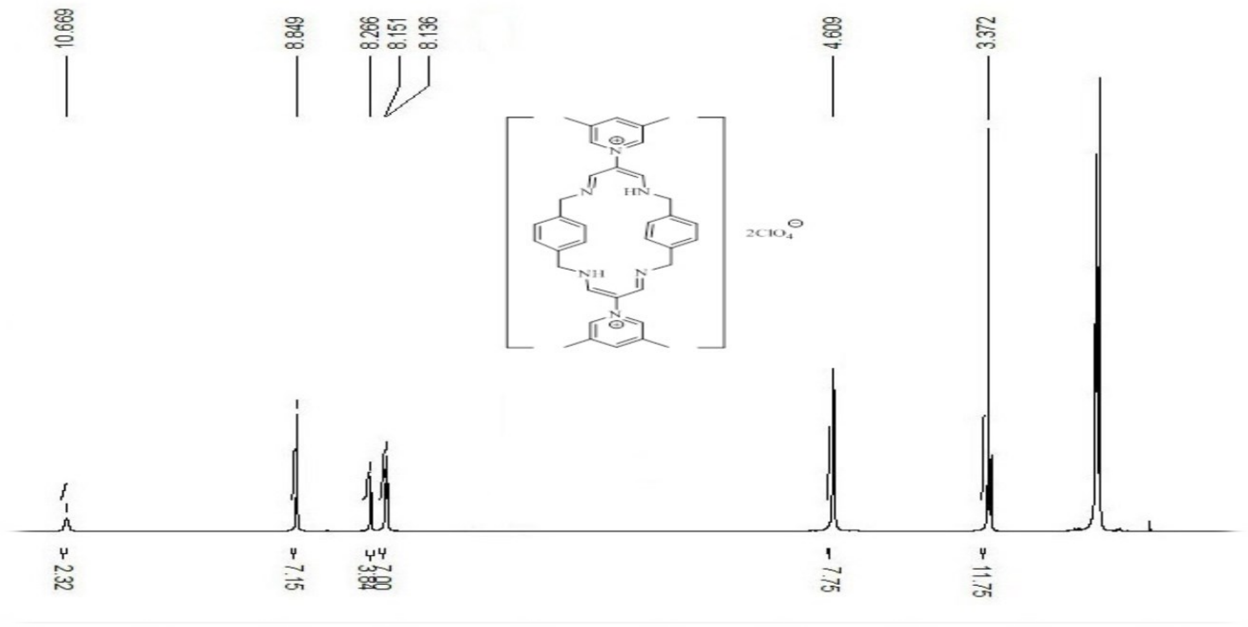

^{1}HNMR of 3,7,11,15-Tetraaza-1,9(1,4)-dibenzenacyclohexadecaphane-3,5,11,13-tetraene-5,13-diyl)bis(3,5-dimethylpyridin-1-ium)-bis(perchlorate) (5b)

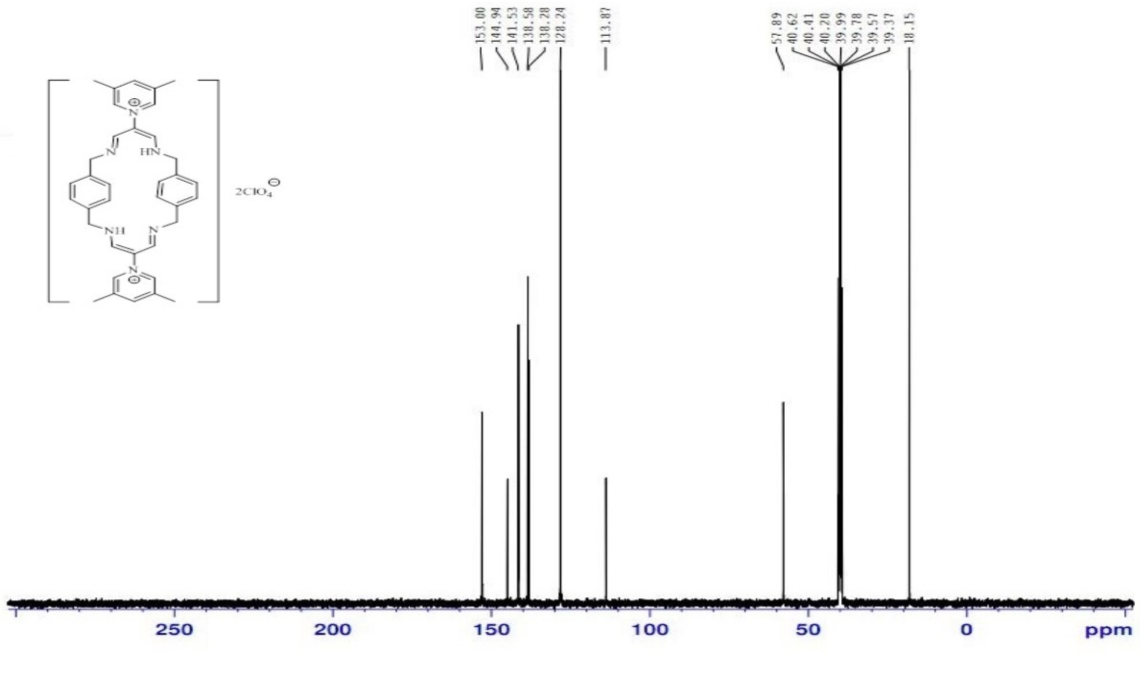

^{13}CNMR of 3,7,11,15-Tetraaza-1,9(1,4)-dibenzenacyclohexadecaphane-3,5,11,13-tetraene-5,13-diyl)bis(3,5-dimethylpyridin-1-ium)-bis(perchlorate) (5b)

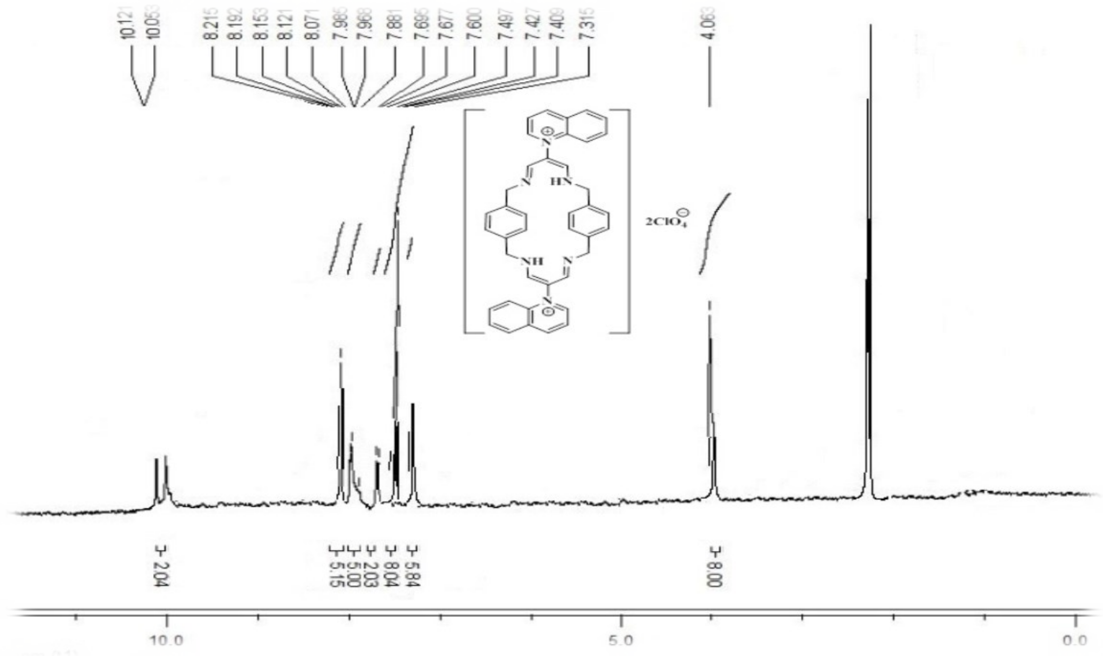

^{1}HNMR of 3,7,11,15-Tetraaza-1,9(1,4)-dibenzenacyclohexadecaphane-3,5,11,13-tetraene-5,13-diyl)bis(quinolin-1-ium)-bis(perchlorate) (5c)

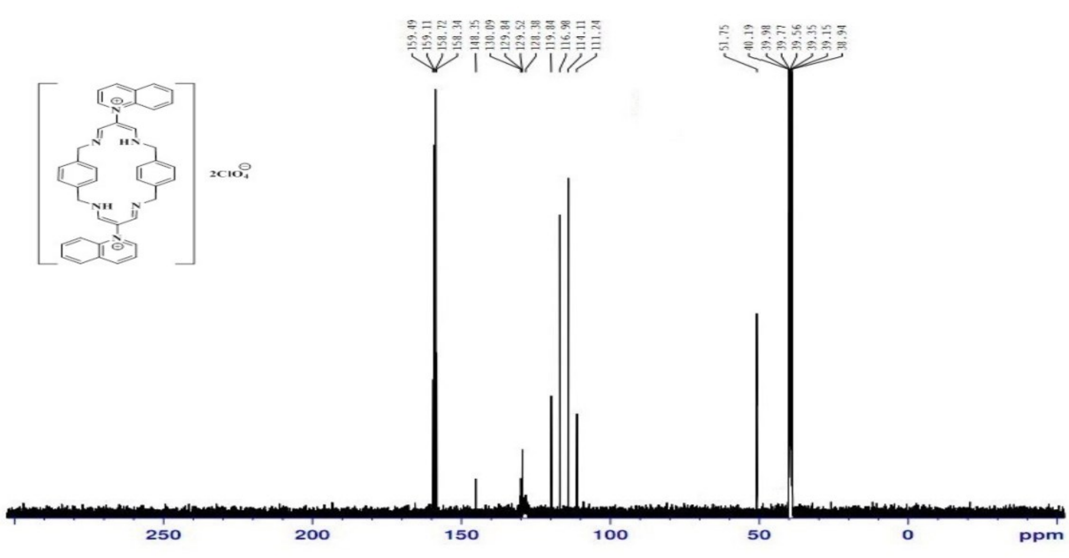

^{13}CNMR of 3,7,11,15-Tetraaza-1,9(1,4)-dibenzenacyclohexadecaphane-3,5,11,13-tetraene-5,13-diyl)bis(quinolin-1-ium)-bis(perchlorate) (5c)

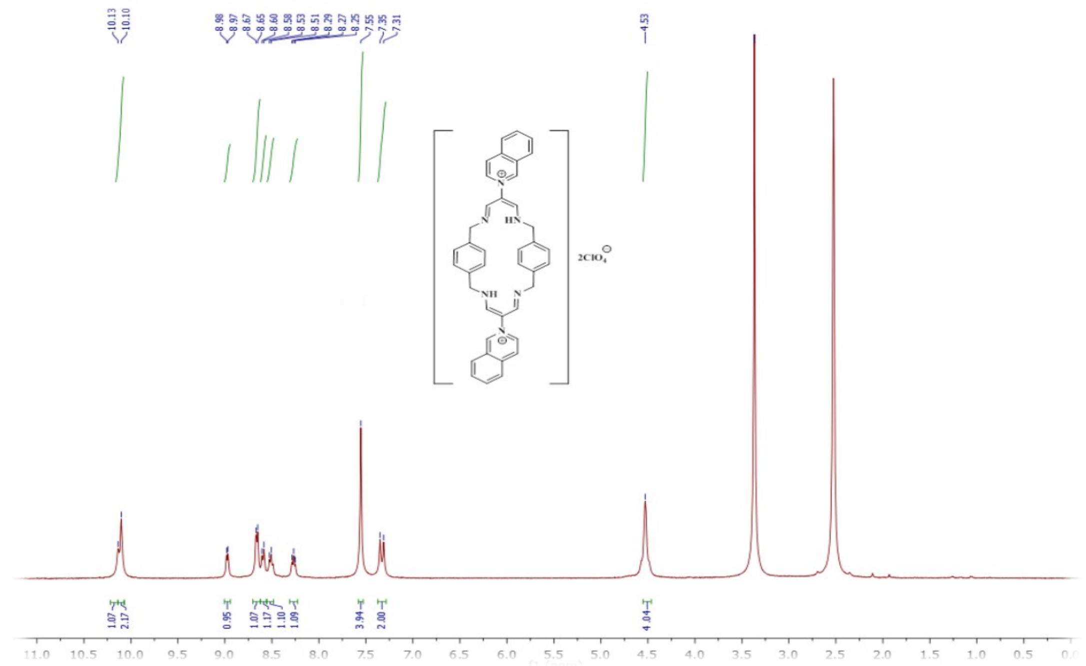

^{1}HNMR of 3,7,11,15-Tetraaza-1,9(1,4)-dibenzenacyclohexadecaphane-3,5,11,13-tetraene-5,13-diyl)bis(isoquinolin-2-ium)-bis(perchlorate) (5d)

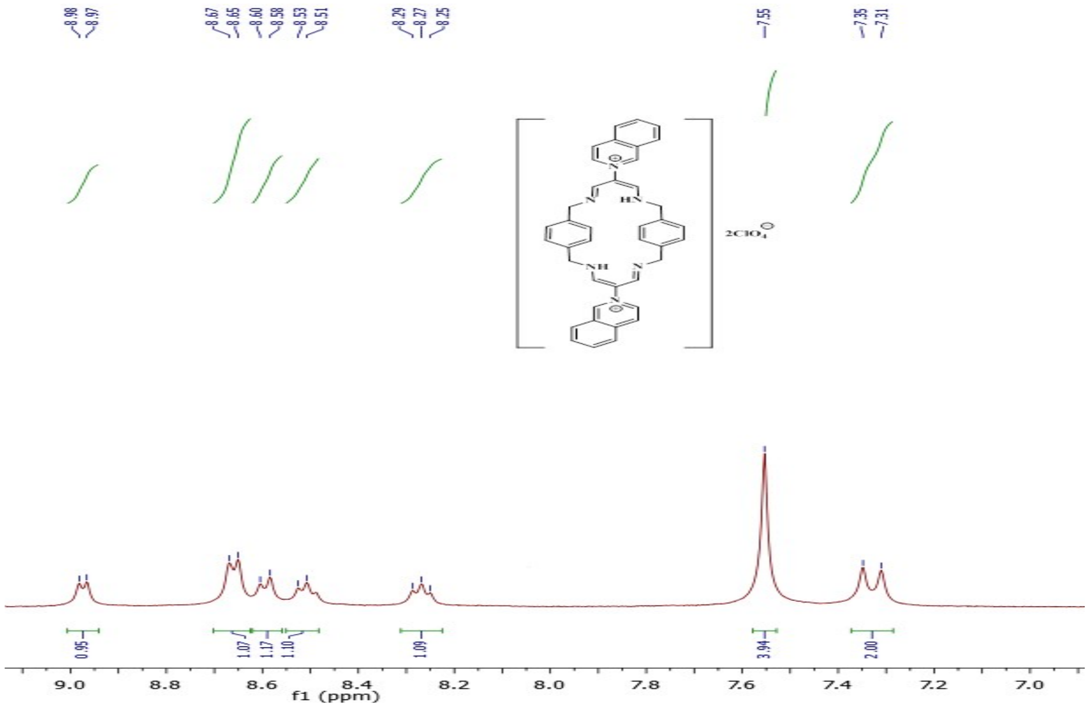

Expand of 3,7,11,15-Tetraaza-1,9(1,4)-dibenzenacyclohexadecaphane-3,5,11,13-tetraene-5,13-diyl)bis(isoquinolin-2-ium)-bis(perchlorate) (5d)

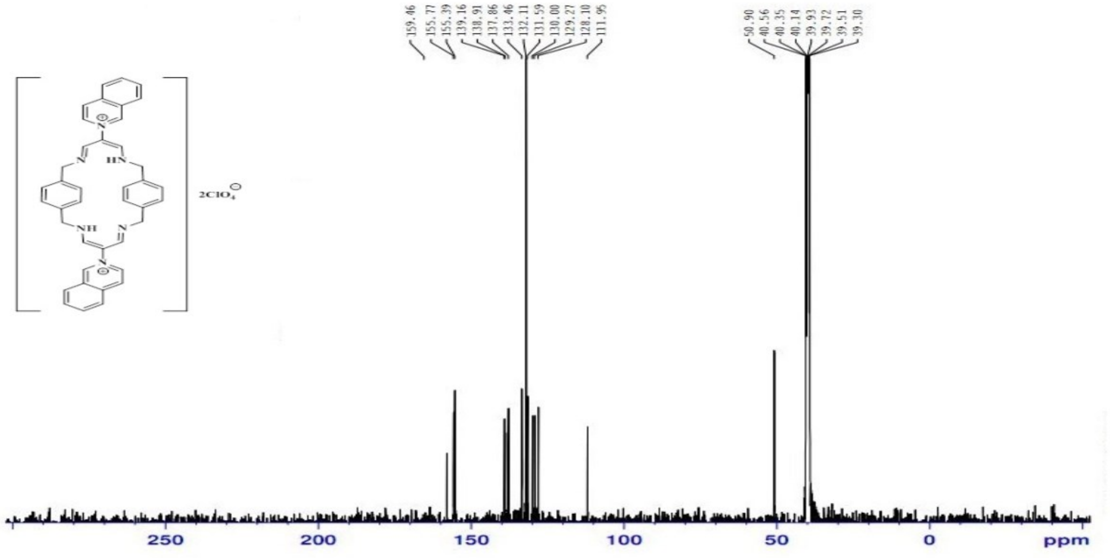

^{13}CNMR of 3,7,11,15-Tetraaza-1,9(1,4)-dibenzenacyclohexadecaphane-3,5,11,13-tetraene-5,13-diyl)bis(isoquinolin-2-ium)-bis(perchlorate) (5d)

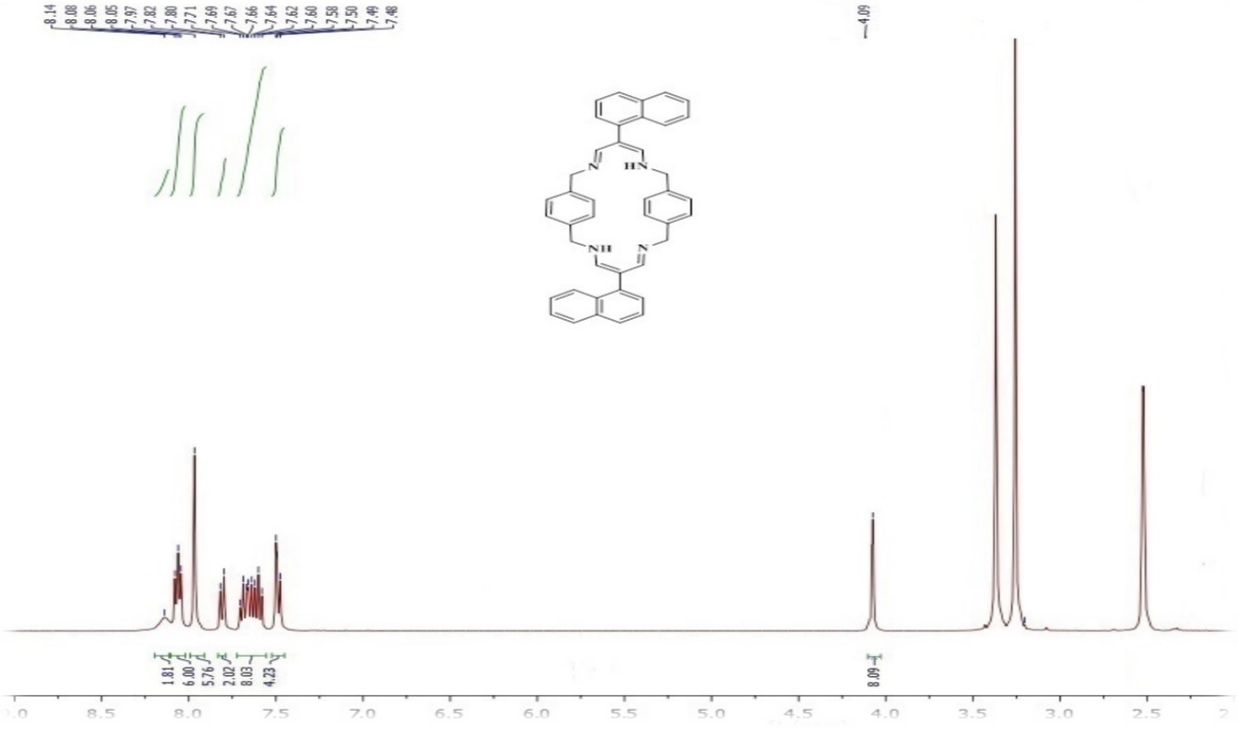

^{1}HNMR of 5,13-Di(naphthalen-1-yl)-3,7,11,15-tetraaza-1,9(1,4) dibenzenacyclohexadecaphane-4,6,12,14-tetraene (5f)

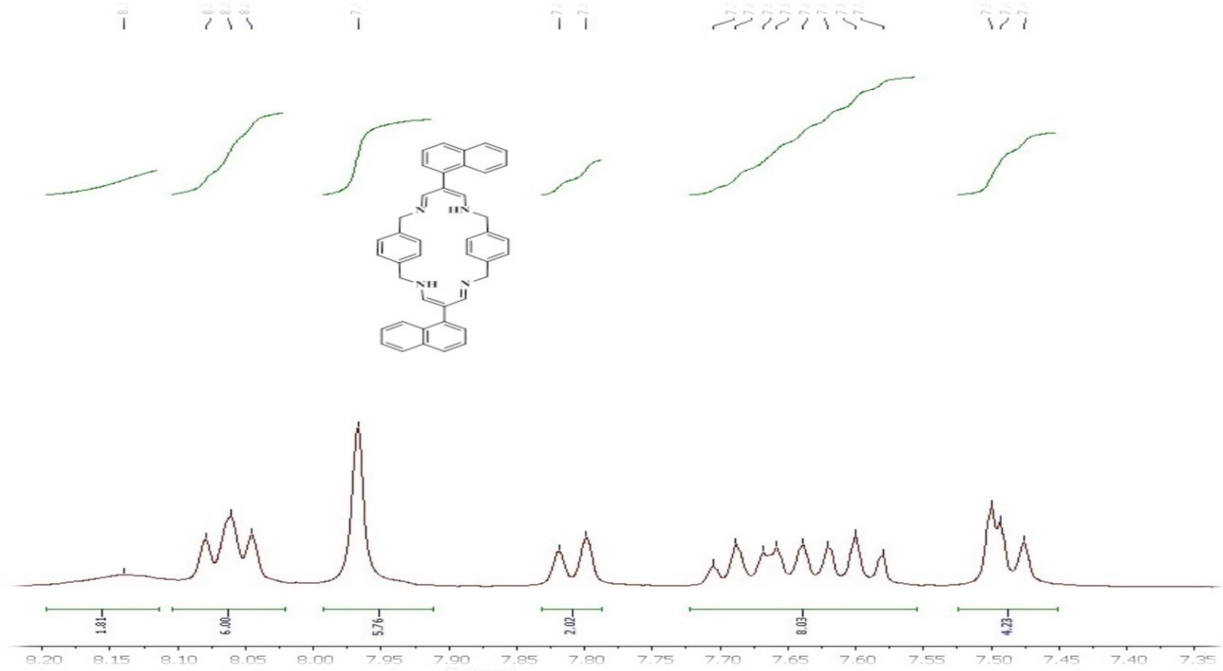

Expand of 5,13-Di(naphthalen-1-yl)-3,7,11,15-tetraaza-1,9(1,4) dibenzenacyclohexadecaphane-4,6,12,14-tetraene (5f)

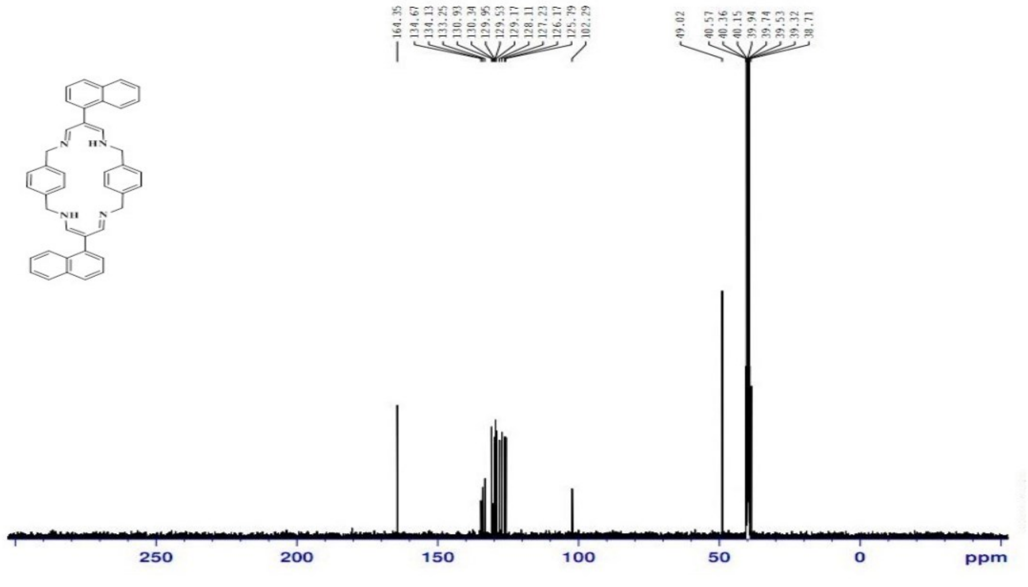

^{13}CNMR of 5,13-Di(naphthalen-1-yl)-3,7,11,15-tetraaza-1,9(1,4) dibenzenacyclohexadecaphane-4,6,12,14-tetraene (5f)

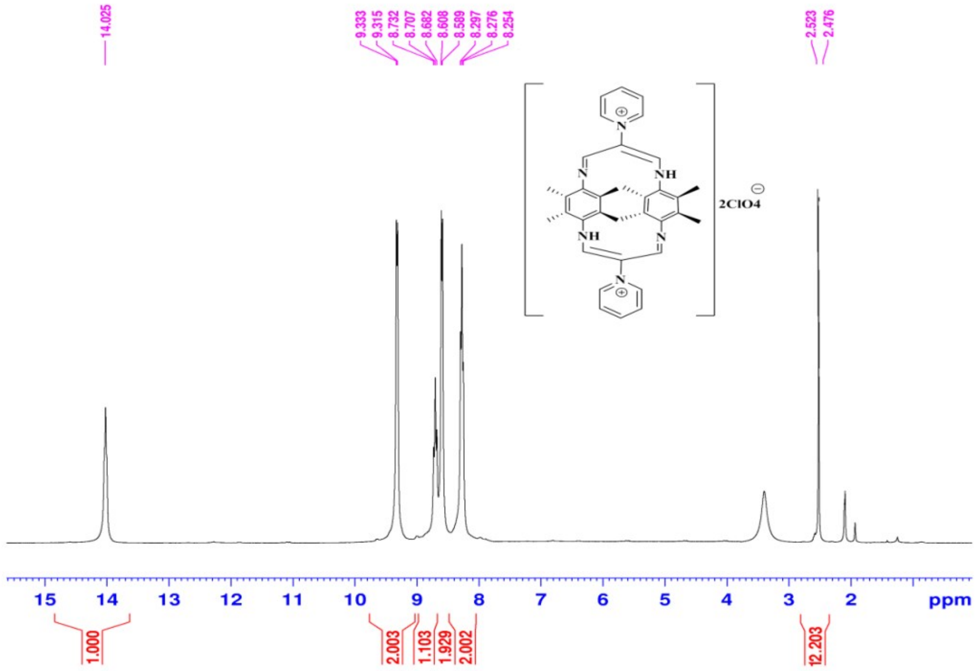

^{1}HNMR of Octamethyl-2,6,8,12-tetraaza-1,7(1,4)-dibenzenacyclododecaphane-3,5,9,11-tetraene-4,10-diyl)bis(pyridin-1-ium)- bis(perchlorate) (6a)

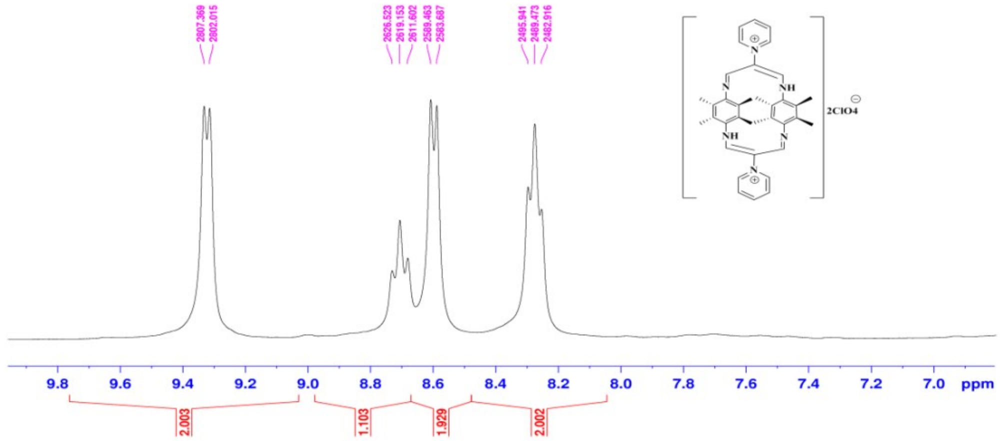

Expand of Octamethyl-2,6,8,12-tetraaza-1,7(1,4)-dibenzenacyclododecaphane-3,5,9,11-tetraene-4,10-diyl)bis(pyridin-1-ium)- bis(perchlorate) (6a)

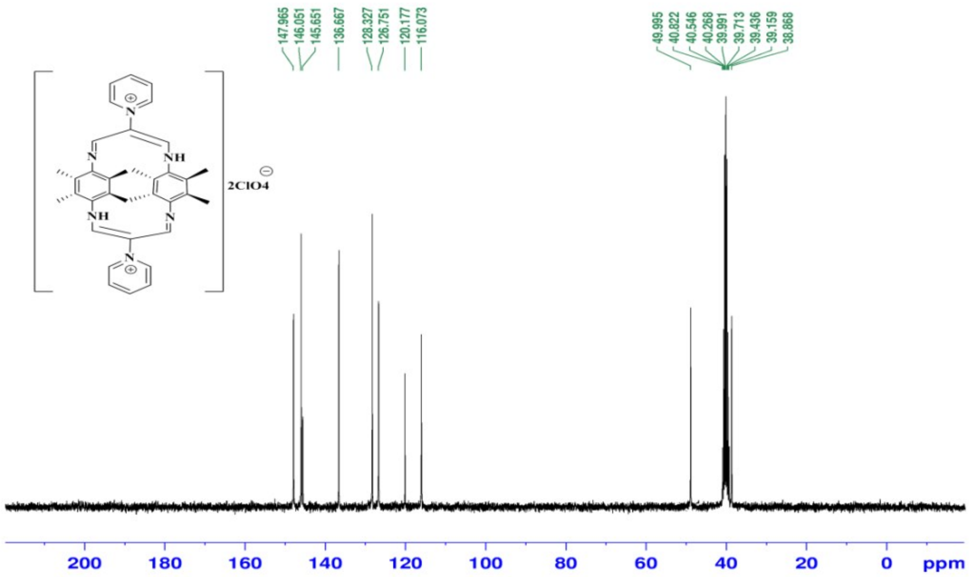

^{13}CNMR of Octamethyl-2,6,8,12-tetraaza-1,7(1,4)-dibenzenacyclododecaphane-3,5,9,11-tetraene-4,10-diyl)bis(pyridin-1-ium)- bis(perchlorate) (6a)

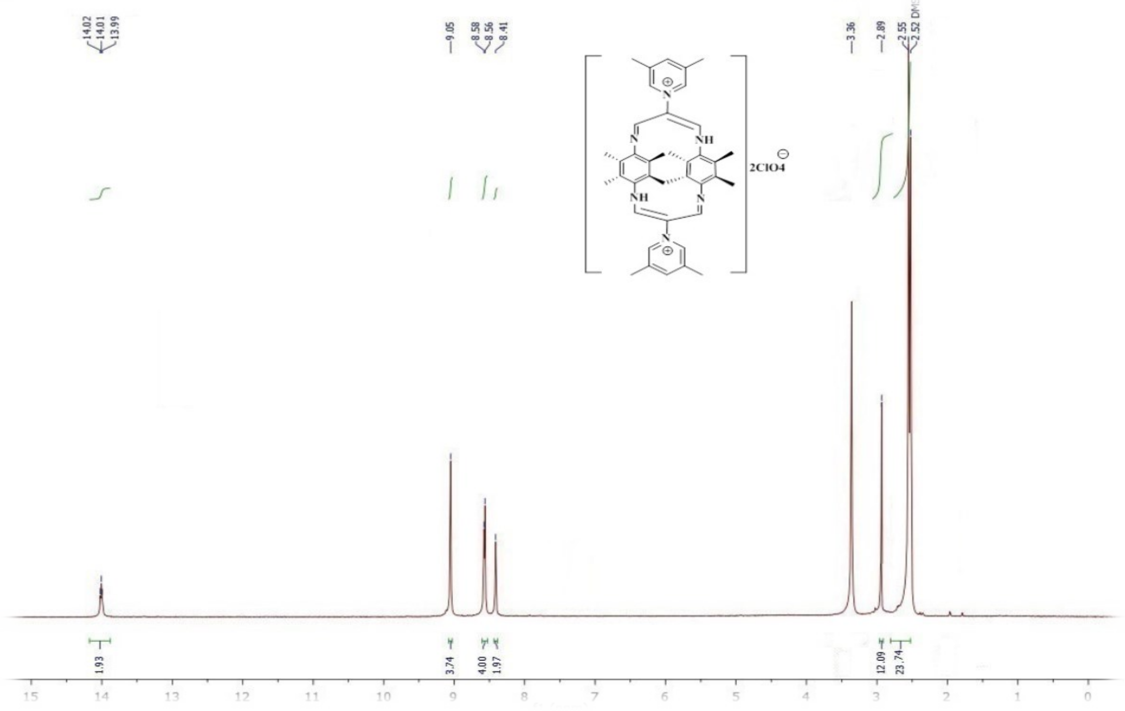

^{1}HNMR of octamethyl-2,6,8,12-tetraaza-1,7(1,4)-dibenzenacyclododecaphane-3,5,9,11-tetraene-4,10-diyl)bis(3,5-dimethylpyridin-1-ium)- bis(perchlorate) (6b)

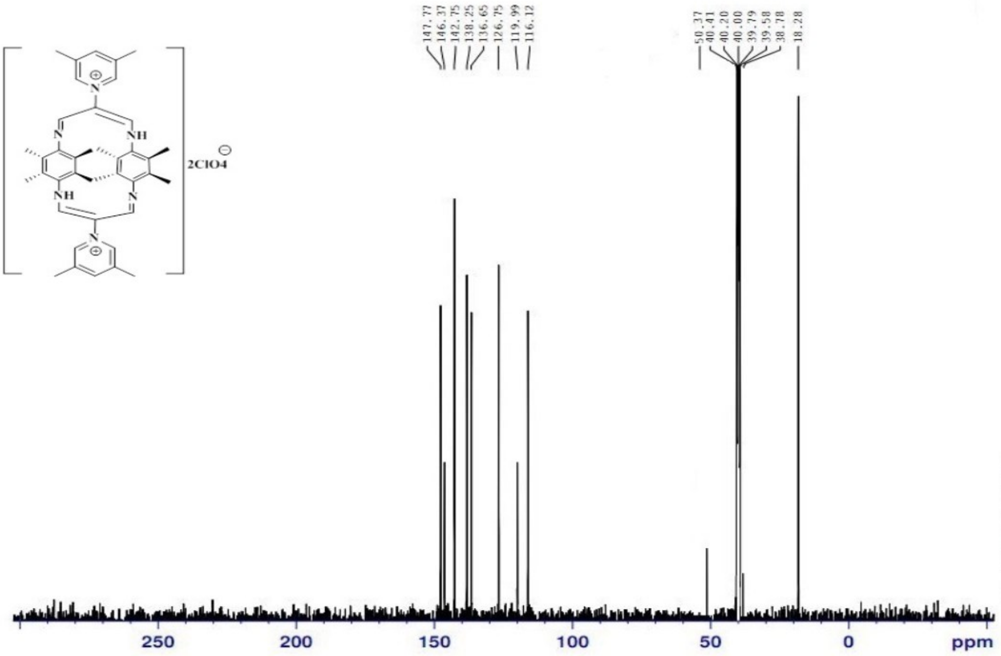

^{13}CNMR of octamethyl-2,6,8,12-tetraaza-1,7(1,4)-dibenzenacyclododecaphane-3,5,9,11-tetraene-4,10-diyl)bis(3,5-dimethylpyridin-1-ium)- bis(perchlorate) (6b)

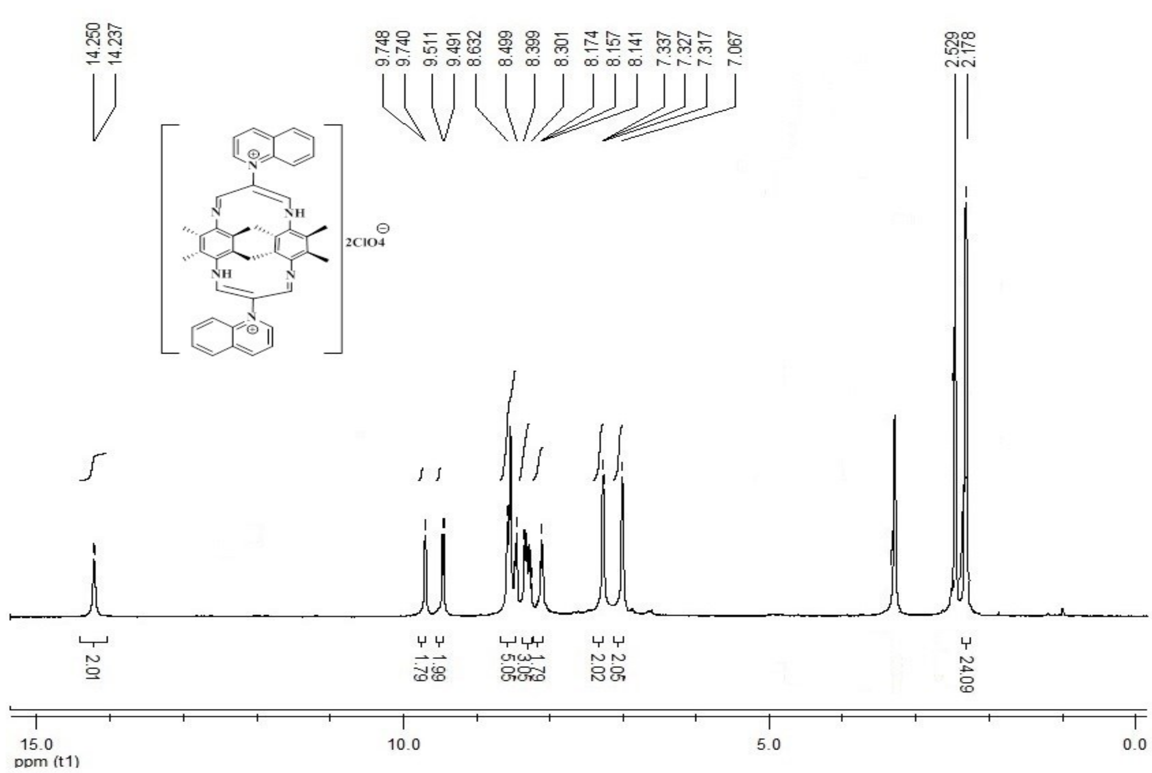

^{1}HNMR of octamethyl-2,6,8,12-tetraaza-1,7(1,4)-dibenzenacyclododecaphane-3,5,9,11-tetraene-4,10-diyl)bis(quinolin-1-ium)-bis(perchlorate) (6c)

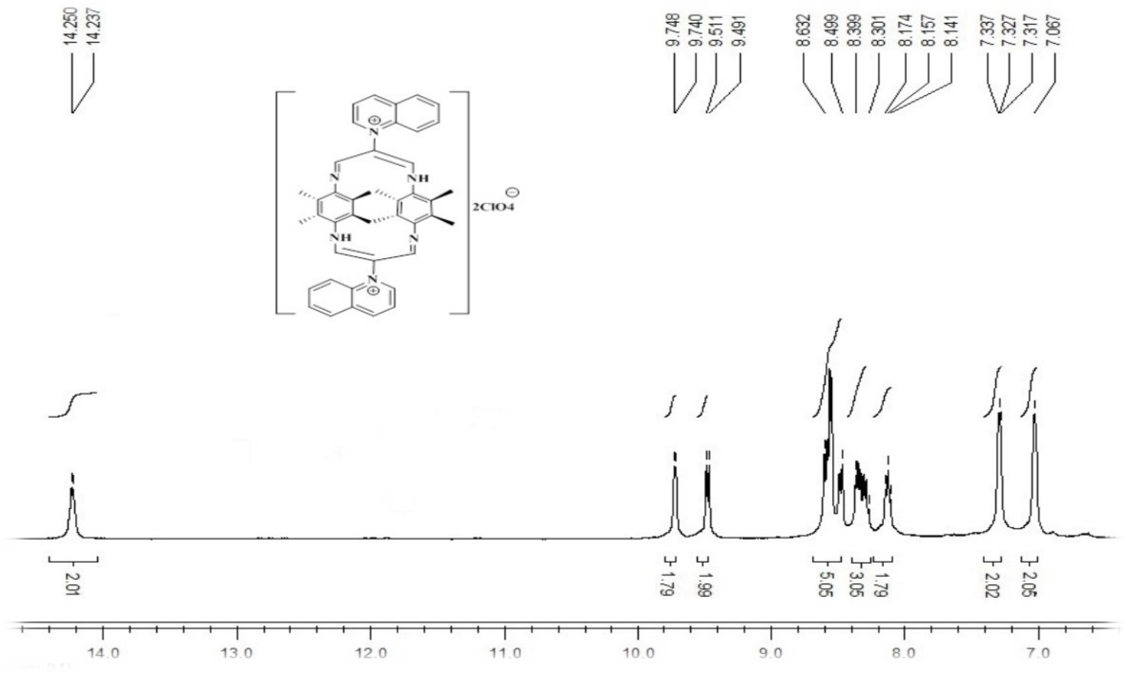

Expand of octamethyl-2,6,8,12-tetraaza-1,7(1,4)-dibenzenacyclododecaphane-3,5,9,11-tetraene-4,10-diyl)bis(quinolin-1-ium)-bis(perchlorate) (6c)

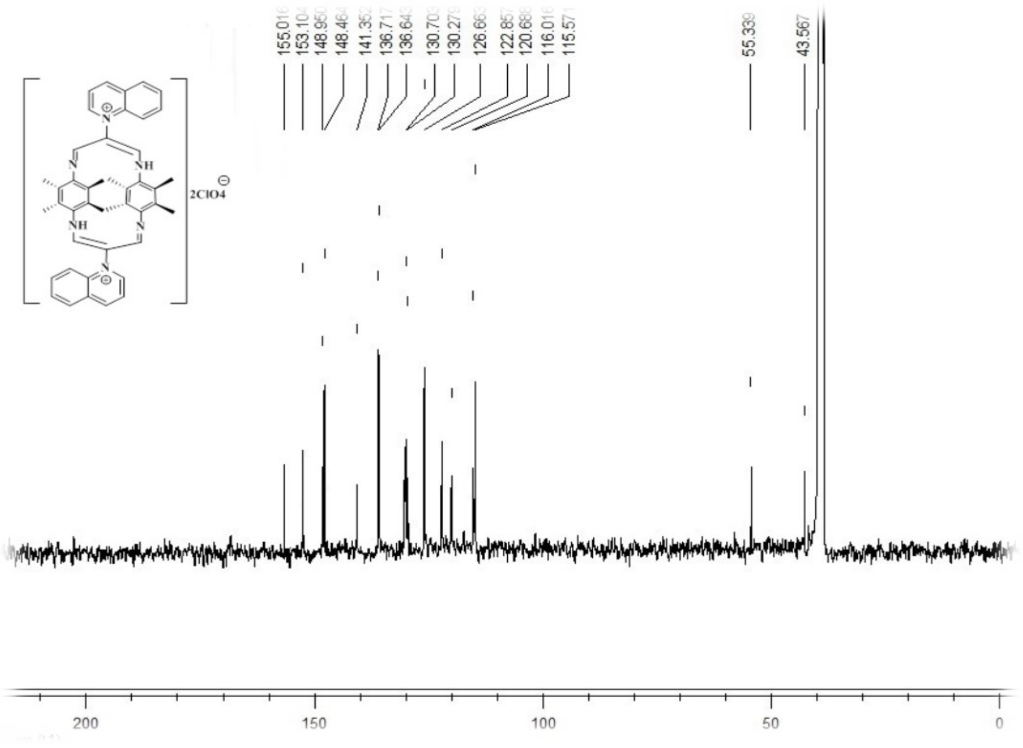

^{13}CNMR of octamethyl-2,6,8,12-tetraaza-1,7(1,4)-dibenzenacyclododecaphane-3,5,9,11-tetraene-4,10-diyl)bis(quinolin-1-ium)-bis(perchlorate) (6c)

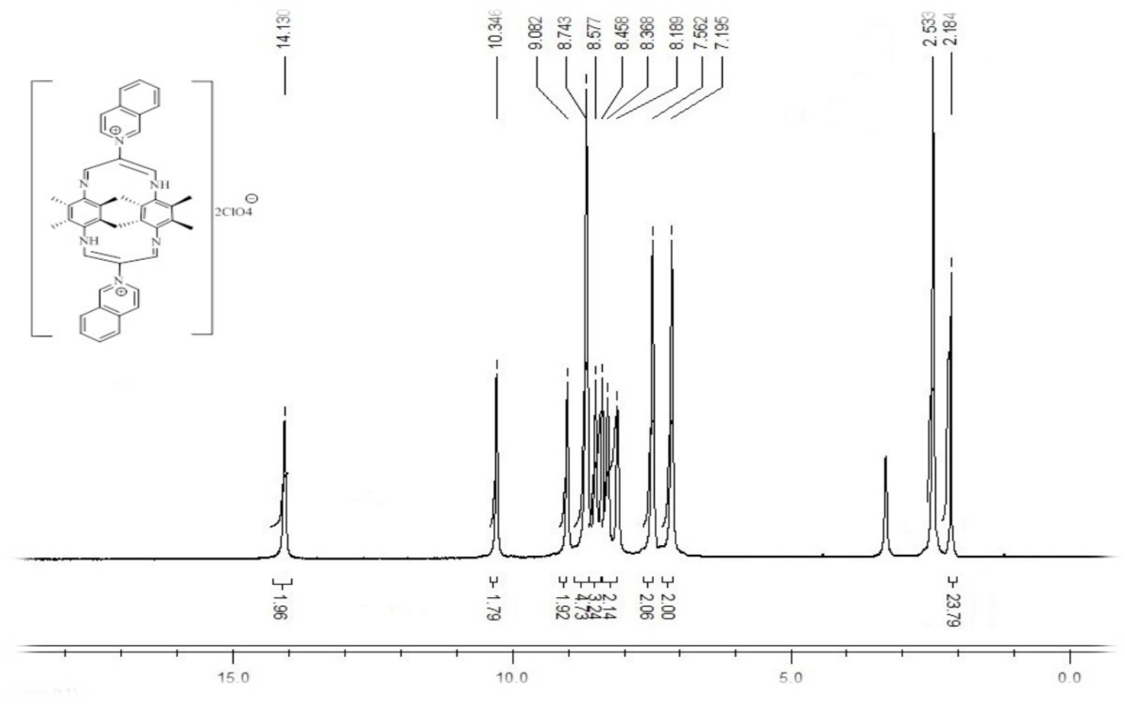

^{1}HNMR of Octamethyl-2,6,8,12-tetraaza-1,7(1,4)-dibenzenacyclododecaphane-3,5,9,11-tetraene-4,10-diyl)bis(isoquinolin-2-ium)-bis(perchlorate) (6d)

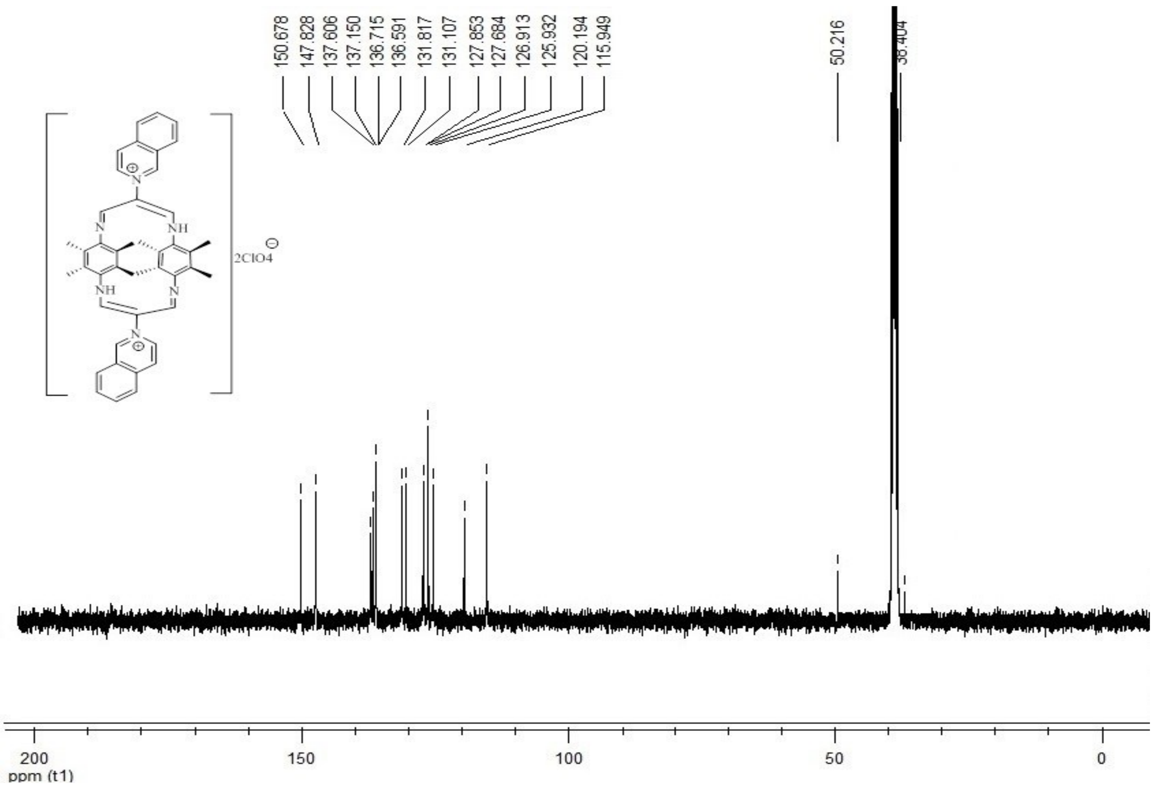

^{13}CNMR of Octamethyl-2,6,8,12-tetraaza-1,7(1,4)-dibenzenacyclododecaphane-3,5,9,11-tetraene-4,10-diyl)bis(isoquinolin-2-ium)-bis(perchlorate) (6d)

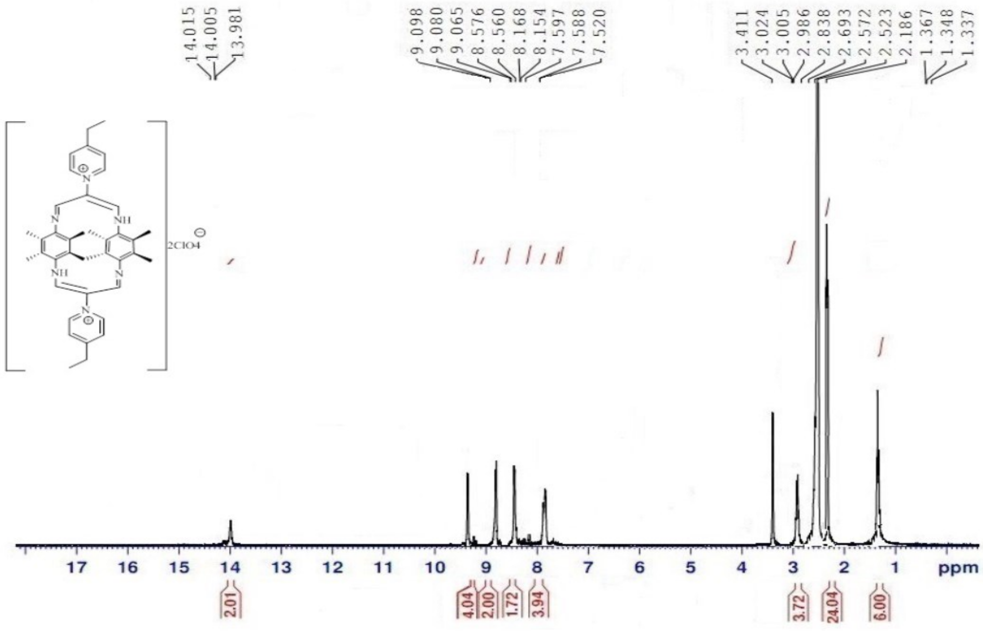

^{1}HNMR of Octamethyl-2,6,8,12-tetraaza-1,7(1,4)-dibenzenacyclododecaphane-3,5,9,11-tetraene-4,10-diyl)bis(4-ethylpyridin-1-ium)-bis(perchlorate) (6e)

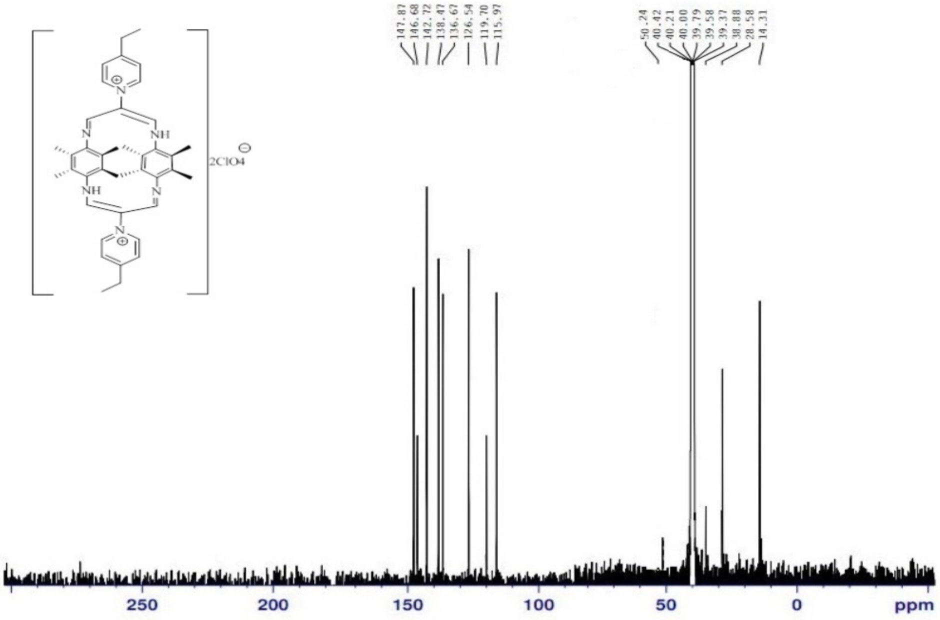

^{13}CNMR of Octamethyl-2,6,8,12-tetraaza-1,7(1,4)-dibenzenacyclododecaphane-3,5,9,11-tetraene-4,10-diyl)bis(4-ethylpyridin-1-ium)-bis(perchlorate) (6e)

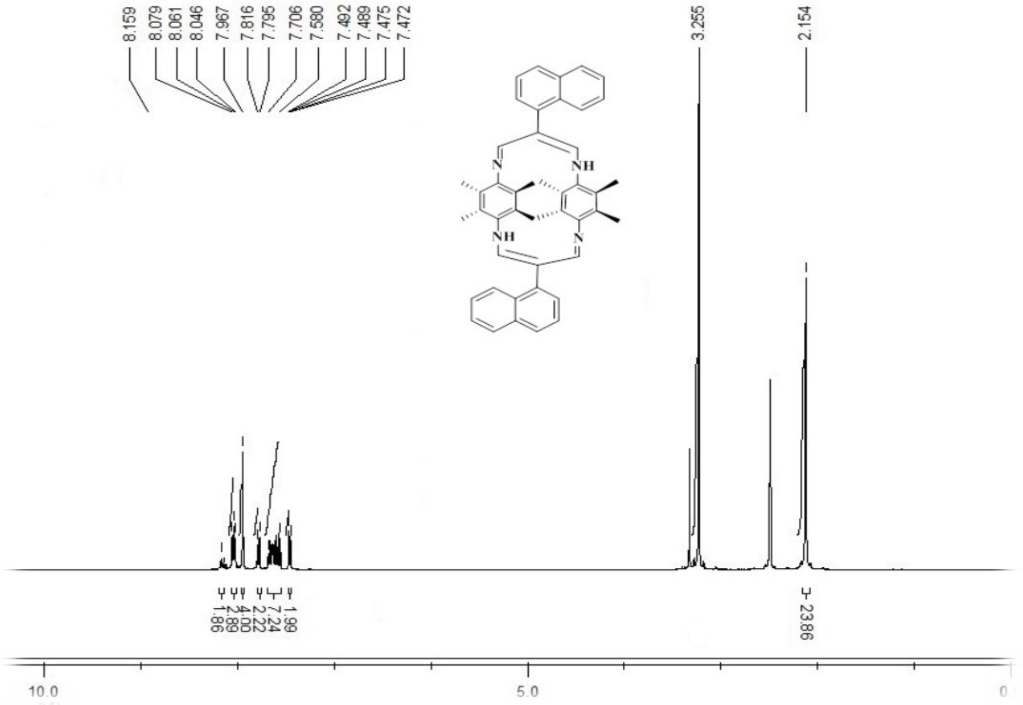

^{1}HNMR of Octamethyl-4,10-di(naphthalen-1-yl)-2,6,8,12-tetraaza-1,7(1,4) dibenzenacyclododecaphane-3,5,9,11-tetraene (6f)

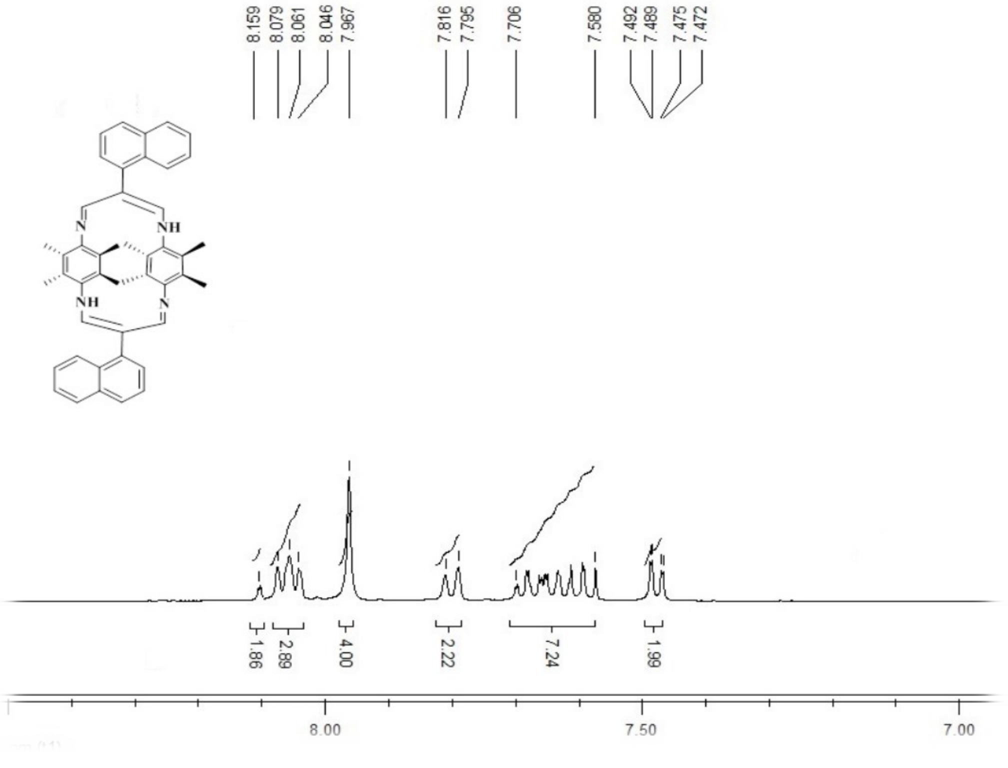

Expand of Octamethyl-4,10-di(naphthalen-1-yl)-2,6,8,12-tetraaza-1,7(1,4) dibenzenacyclododecaphane-3,5,9,11-tetraene (6f)

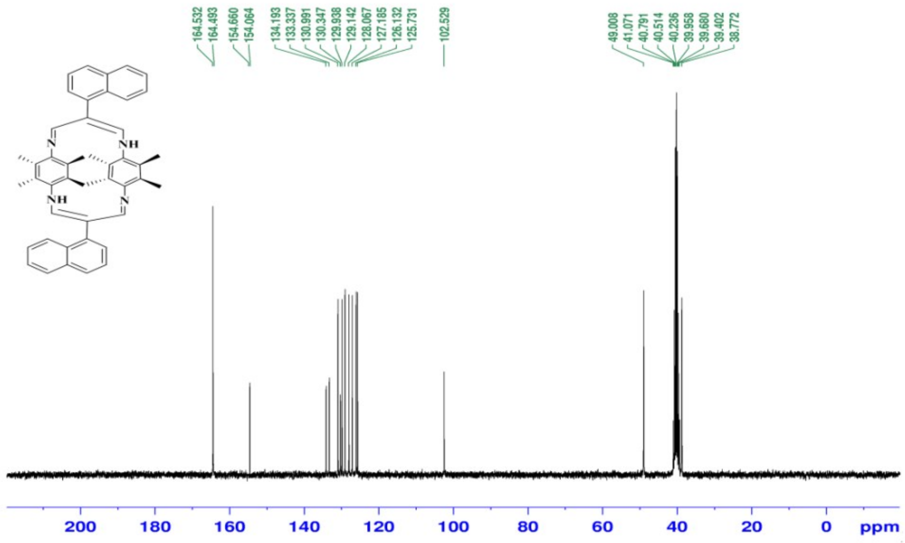

¹³CNMR of Octamethyl-4,10-di(naphthalen-1-yl)-2,6,8,12-tetraaza-1,7(1,4) dibenzenacyclododecaphane-3,5,9,11-tetraene (6f)

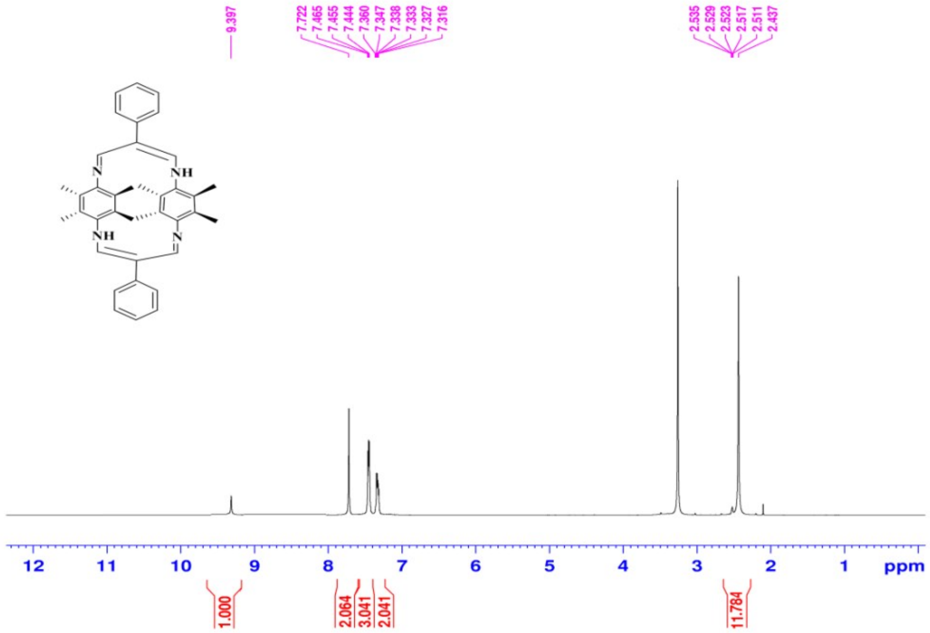

¹HNMR of Octamethyl-4,10-diphenyl-2,6,8,12-tetraaza-1,7(1,4)-dibenzenacyclododecaphane-3,5,9,11-tetraene (6g)

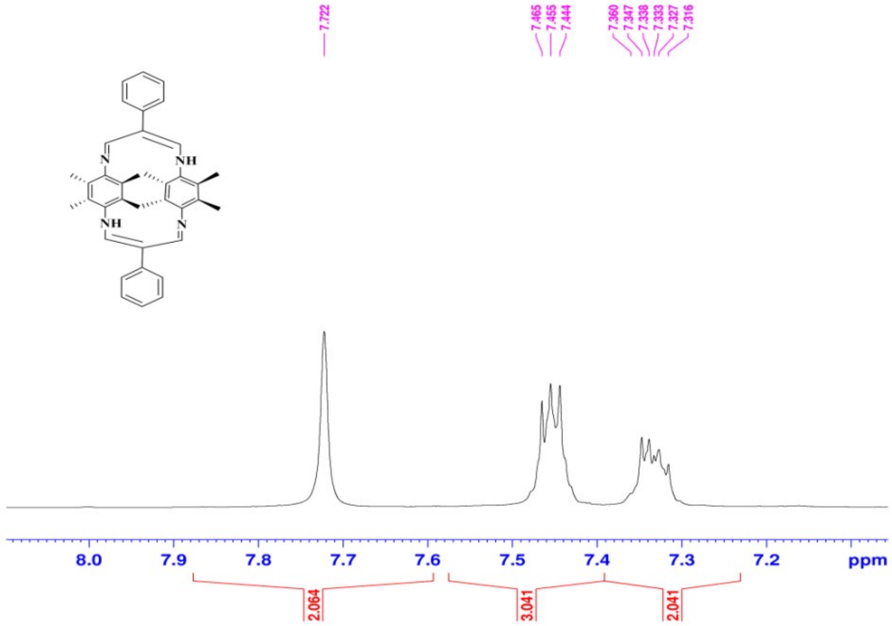

Expand of Octamethyl-4,10-diphenyl-2,6,8,12-tetraaza-1,7(1,4)-dibenzenacyclododecaphane-3,5,9,11-tetraene (6g)

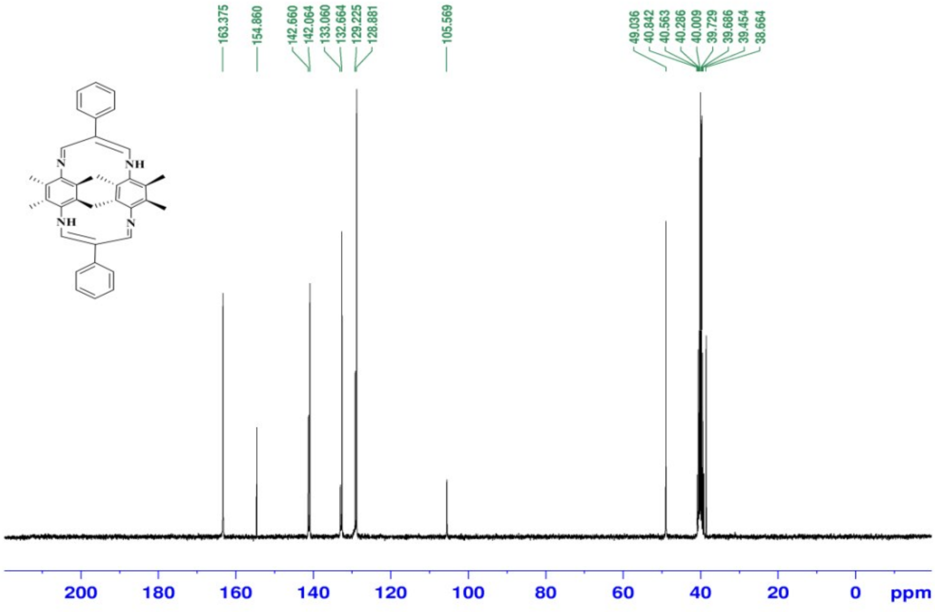

^{13}CNMR of Octamethyl-4,10-diphenyl-2,6,8,12-tetraaza-1,7(1,4)-dibenzenacyclododecaphane-3,5,9,11-tetraene (6g)
